# Integrated microbiome and metabolome profiling in prepubertal girls with vulvar lichen sclerosus

**DOI:** 10.1128/spectrum.04058-25

**Published:** 2026-08-27

**Authors:** Huihui Gao, Fengxia Li, Hongyu Chen, Siming Wu, Junwen Zhang, Chaoqun Wang, Qiuxiang Shen, Li Zhu, Dandan Chen, Lingling Jin, Jie Liu, Jiayu Shen, Liying Sun, Lan Yu

**Affiliations:** 1 Department of Pediatrics and Adolescent Gynecology, Children' s Hospital, Zhejiang University School of Medicine, National Clinical Research Center for Children and Adolescents' Health and Diseases 26441, Hangzhou, China; 2 Zhejiang-Hong Kong China International Joint Laboratory on Reproductive Health of Children and Adolescents, Children' s Hospital, Zhejiang University School of Medicine, National Clinical Research Center for Children and Adolescents' Health and Diseases, Hangzhou, China; 3 Zhejiang Key Laboratory of Neonatal Diseases, Hangzhou, China; 4 Children’s Hospital, Zhejiang University School of Medicine, National Clinical Research Center for Children and Adolescents' Health and Diseases, Hangzhou, China; Xi'an Jiaotong-Liverpool University, Suzhou, Jiangsu Province, China

**Keywords:** vulvar lichen sclerosus, 16S rRNA sequence, metabolomics, 12,13-DHOME, biomarkers

## Abstract

Vulvar lichen sclerosus (VLS) is a chronic anogenital disease with long-term risks of genital disfigurement and squamous cell cancer. The involvement of the gut-skin axis in prepubertal VLS remains unclear. We conducted a cross-sectional study of 43 prepubertal VLS patients and 46 healthy controls, performing fecal 16S rRNA sequencing and serum LC-MS to characterize gut microbiome–metabolome associations and identify altered microbial and metabolic signatures. We further assessed their exploratory classification performance using random forest models. No significant differences in alpha or beta diversity were observed between groups. At the nominal significance level, an increase in *Firmicutes_A* (phylum; *P* = 0.021) and *Streptococcus* (genus; *P* = 0.046) and a decrease in *Phocaeicola_A_858004* (genus; *P* = 0.004), *Bacteroides_H* (genus; *P* = 0.033), and *Streptococcus* (genus; *P* = 0.046) were observed in the VLS group. After multiple testing correction, only *Phocaeicola_A_858004* remained significant (BH-adjusted *q* = 0.041). Linear discriminant analysis effect size identified 23 discriminant taxa, with 12 enriched in VLS patients and 11 enriched in controls. Functional prediction suggested perturbations in amino acid and lipid metabolism, aligning with host metabolic pathways. Metabolomic analysis identified 123 dysregulated metabolites, with integrative analysis showing correlations between key bacteria (e.g., *s_Porphyromonas_A_859426_somerae*) and metabolites (e.g., 12,13-DHOME). Furthermore, serum metabolites showed strong discriminative performance (area under the curve [AUC] = 0.93), compared with microbial features alone (AUC = 0.83) or combined models (AUC = 0.87). These findings indicate modest but distinctive associations between gut microbiome and host serum metabolic profiles in girls with VLS and suggest that integrated microbial and metabolic signatures may serve as exploratory non-invasive biomarkers for this condition.

**IMPORTANCE:**

Integrating gut microbiome and serum metabolome analyses, this study characterized gut-skin axis associations in prepubertal vulvar lichen sclerosus (VLS). Overall gut microbial diversity and community structure were preserved without statistically significant differences after multiple testing correction. However, linear discriminant analysis effect size identified discriminative taxa between groups, suggesting potential compositional differences at the feature level. In contrast, serum metabolomic profiles showed more robust alterations in VLS patients. The microbial and metabolic signatures demonstrated strong discriminative ability between VLS and control groups, supporting their potential as exploratory non-invasive biomarkers. These results indicate that modest gut microbiome–metabolome perturbations are early pathogenic events in VLS, preceding overt microbial structural remodeling, providing a reference for further exploration of early detection strategies in this condition. These findings suggest that VLS-related perturbations may be more evident at the metabolic than at the gut microbiome taxonomic level, providing a framework for future studies of gut-skin interactions and early disease characterization.

## INTRODUCTION

Vulvar lichen sclerosus (VLS) is a chronic, inflammatory skin disease primarily affecting the anogenital region (1). It occurs in approximately 0.1% of prepubertal girls and up to 3% of postmenopausal women (2). The main clinical manifestations include vulvar pruritus, burning, tingling, and pain. In severe cases, patients may experience skin fissures with bleeding, constipation, and dysuria (3). Untreated VLS can lead to sexual and urinary dysfunction, genital disfigurement, and increased risk of squamous cell cancer (4, 5).

The etiology and pathogenesis remain unclear, but autoimmune involvement has been suggested (6). Previous studies have shown that 15%–34% of adult women and 14% of girls with VLS have coexisting allergies or autoimmune diseases (7). Dysbiosis of the skin and gut microbiota has been implicated in various dermatological conditions (8), and such an imbalance in microbiota composition may contribute to immune dysfunction and disease development (9). Notably, a recent study has suggested that intestinal dysbiosis may be involved in the pathogenesis of VLS, although further research is needed to confirm this association (10).

The gut microbiome plays essential roles from early life, including protection against pathogens, maintenance of metabolic homeostasis, and regulation of immune development (11). It also influences skin health through the gut-skin axis, a bidirectional communication system in which gut microbial communities modulate systemic and cutaneous immune responses. Previous studies have shown that changes in gut microbial communities can trigger an immunological response, resulting in skin diseases such as allergies, acne vulgaris, atopic dermatitis, alopecia areata, rosacea, and hidradenitis suppurativa (12). Based on this accumulating evidence, we hypothesize that gut microbiome alteration may contribute significantly to the pathogenesis of VLS through immune-mediated mechanisms.

To date, few studies have investigated the gut microbiota in prepubertal girls with VLS. Most of them focused on adults or lacked integrated functional analyses. Therefore, we performed an integrated study combining 16S rRNA sequencing and serum metabolomics to characterize the gut microbiota and systemic metabolism in prepubertal girls with VLS and healthy controls. Our aims were to identify distinct microbial and metabolic signatures, explore potential correlated changes, and assess their exploratory diagnostic value, thereby providing preliminary evidence for understanding VLS pathophysiology and future research directions.

## RESULTS

### Microbiome diversity and composition of VLS

A total of 89 participants (43 VLS patients and 46 healthy controls) were initially enrolled. After quality control (QC), fecal samples from 75 participants (42 VLS and 33 controls) were successfully sequenced for the 16S rRNA gene, and serum samples from 81 participants (39 VLS and 42 controls) were profiled by LC-MS. Paired fecal and serum samples were available for 67 participants (38 VLS and 29 controls) for integrative analysis (File S1). The demographic and clinical characteristics of the study population are summarized in File S2. In the VLS group, the median age was 6.1 years (interquartile range [IQR]: 4.8–8.9, range: 3.3–13.3 years), and the median body weight was 20.0 kg (IQR: 17.5–28.5 kg). In the control group, the median age was 10.1 years (IQR: 7.5–12.1, range: 3.1–15.4 years), and the median body weight was 31.3 kg (IQR: 23.0–44.0 kg). The median disease duration in VLS patients was 6.0 months (IQR: 1.0–12.0), ranging from 2 days to 6 years.

As for 16S sequencing, after demultiplexing in QIIME2, a total of 4.1 million reads were obtained, with an average of 54,114 reads per sample. Rarefaction curves approached saturation for all samples, indicating sufficient sequencing depth to detect rare amplicon sequence variants (ASVs) (File S3A and B).

**Fig 1:**
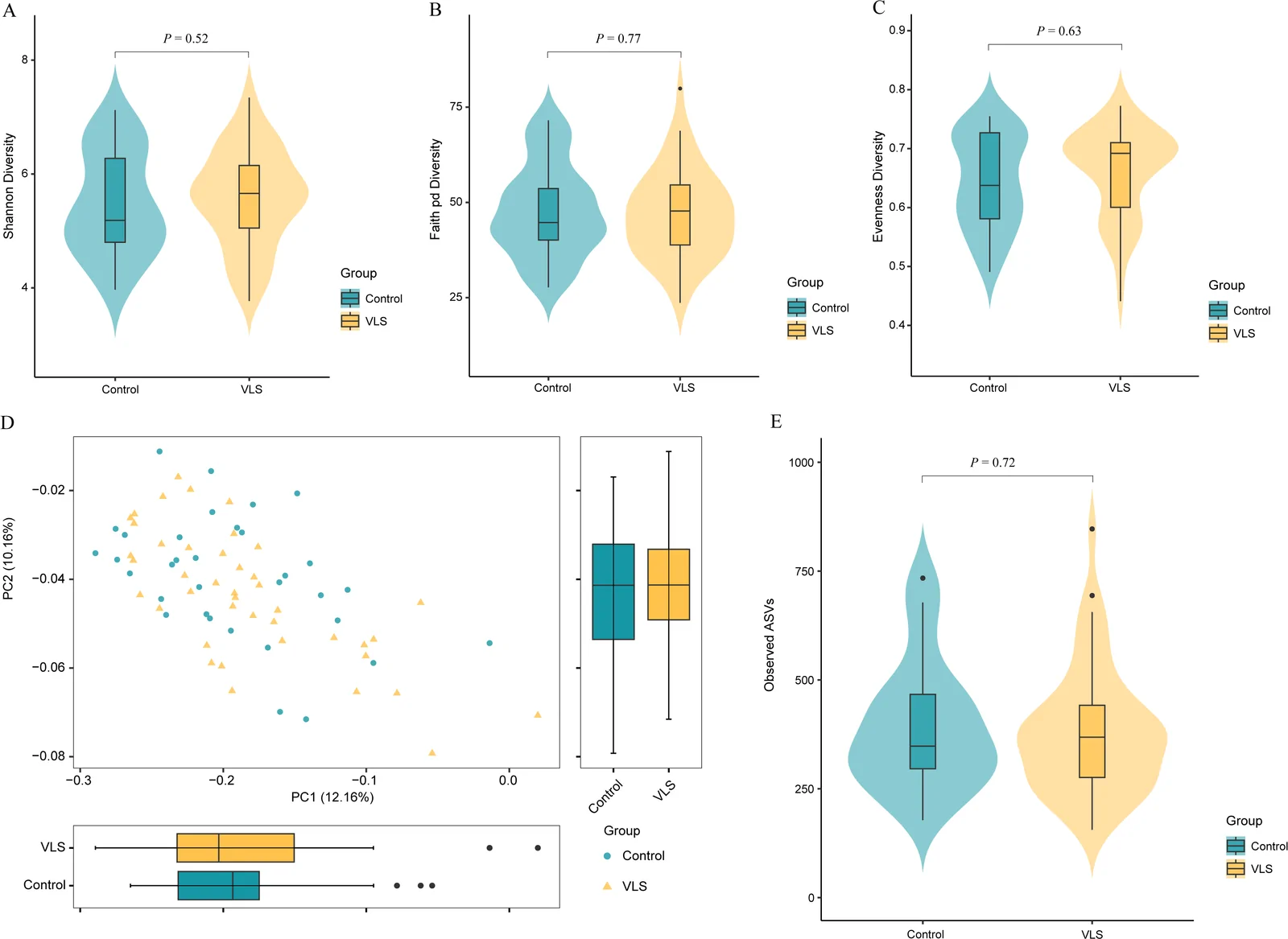
Microbiome diversity analysis between control and VLS groups. (**A–C**) Boxplots of alpha diversity metrics, including Shannon index (**A**), Faith’s phylogenetic diversity (**B**), and Evenness (**C**), between control and VLS groups. No significant differences were observed between groups. (**D**) PCoA based on unweighted UniFrac distances. The percentage of variation explained by each axis is shown in parentheses. Each point represents a sample, colored by group (control or VLS). No clear separation between groups was detected. (**E**) Boxplot of observed ASVs before rarefaction (raw ASV counts) in the control and VLS groups. No significant difference in species richness was detected between groups.

Alpha diversity metrics, including Shannon index, Faith’s phylogenetic diversity, and Evenness, revealed no significant differences between the VLS and control groups (Shannon: *P* = 0.72, Faith’s phylogenetic diversity [Faith PD]: *P* = 0.52, Evenness: *P* = 0.63; Fig. 1A through C). Principal Coordinate Analysis (PCoA) based on unweighted UniFrac dissimilarity showed no significant separation of overall microbial community structure between groups (Fig. 1D). Similarly, species richness, as measured by observed ASVs, was comparable between VLS and control groups (Fig. 1E).

**Fig 2:**
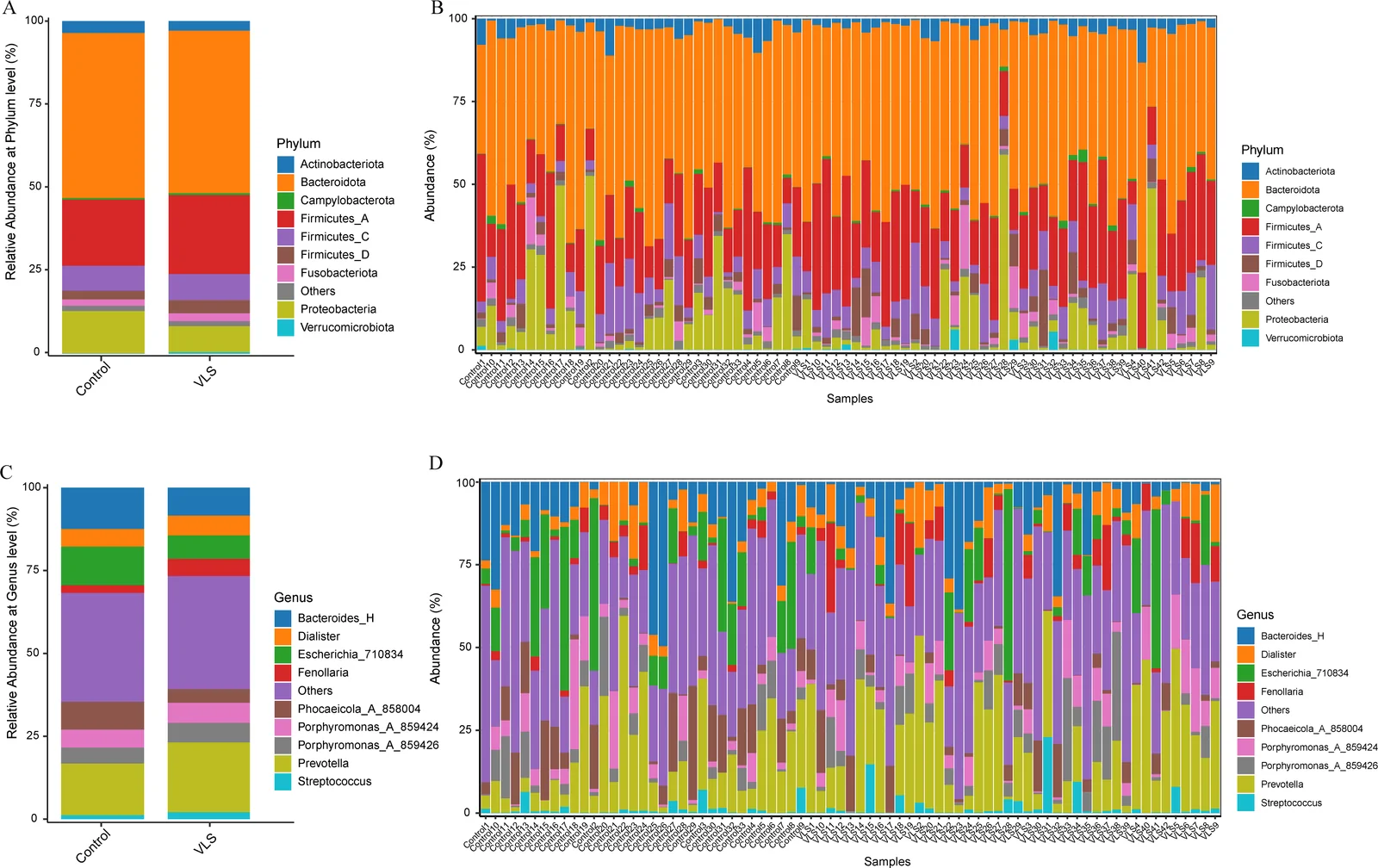
Taxonomic composition of gut microbiota at phylum and genus levels. (**A and C**) Relative abundance of major bacterial phyla (**A**) and genera (**C**) in control and VLS groups. The *y*-axis represents relative abundance. *Bacteroidota* and *Firmicutes_A* were the predominant phyla in both groups at the phylum level (**A**). *Prevotella*, *Bacteroides_H*, and *Escherichia_710834* were among the most abundant genera (**C**). (**B and D**) Stacked bar plots depicting taxonomic profiles at the phylum (**B**) and genus (**D**) levels across groups. Each bar represents one sample, with colors indicating different taxa as shown in the legends. Sample IDs are listed on the *x*-axis.

At the phylum level, both groups were dominated by *Bacteroidota*, *Firmicutes* (*Firmicutes_A*, *Firmicutes_C*), and *Proteobacteria*, followed by *Firmicutes_D*, *Actinobacteriota*, *Fusobacteriota*, *Campylobacterota*, and *Verrucomicrobiota* (Fig. 2A and B; File S4). Although an increased abundance of *Firmicutes_A* (VLS: 0.2374 versus control: 0.1991; raw *P* = 0.021) was observed, the difference did not remain statistically significant after Benjamini-Hochberg (BH) correction for multiple comparisons (*Firmicutes_A*, BH-adjusted *q* = 0.213). The complete list of *P* values and adjusted *q* values is provided in File S4. At the genus level, the most abundant taxa included *Prevotella*, *Bacteroides_H*, *Escherichia_710834*, *Dialister*, *Fenollaria*, *Phocaeicola_A_858004*, *Porphyromonas_A_859424*, *Porphyromonas_A_859426*, and *Streptococcus* (Fig. 2C and D, Table 1). Among these, *Phocaeicola_A_858004* showed a significant decrease in the VLS group compared to the control group (raw *P* = 0.0041, Wilcoxon test, BH-adjusted *q* = 0.041). *Bacteroides_H* and *Streptococcus* showed a minor difference in VLS patients (raw *P* = 0.033 and *P* = 0.046, respectively), but these differences were not reached statistically significant after correction.

**TABLE 1:** Relative abundance of microbial communities at genus

Genus	Mean relative abundance		*P* value	Adjusted *q* value
	VLS	Control		
*Bacteroides_H*	0.084645	0.124729	**0.033071** ^*a*^	0.136890
*Dialister*	0.059289	0.052974	0.091900	0.183800
*Escherichia_710834*	0.07107	0.117026	0.131583	0.219305
*Fenollaria*	0.051896	0.023402	0.054756	0.136890
*Phocaeicola_A_858004*	0.040965	0.083573	**0.004099** ^*a*^	**0.040990** ^*a*^
*Porphyromonas_A_859424*	0.060819	0.054503	0.502885	0.502885
*Porphyromonas_A_859426*	0.058876	0.048215	0.305923	0.382404
*Prevotella*	0.210779	0.155523	0.194539	0.277913
*Streptococcus*	0.020569	0.011909	**0.046134** ^*a*^	0.136890
Others	0.3410857	0.328140	0.415180	0.461311

^*a*^ *P* values were calculated via the Wilcoxon rank-sum test, with significance set at *P* < 0.05, bold font denotes statistical significance. BH correction was used for multiple-testing adjustment.

### Essential microbes and microbial predicted functions in relation to VLS

**Fig 3:**
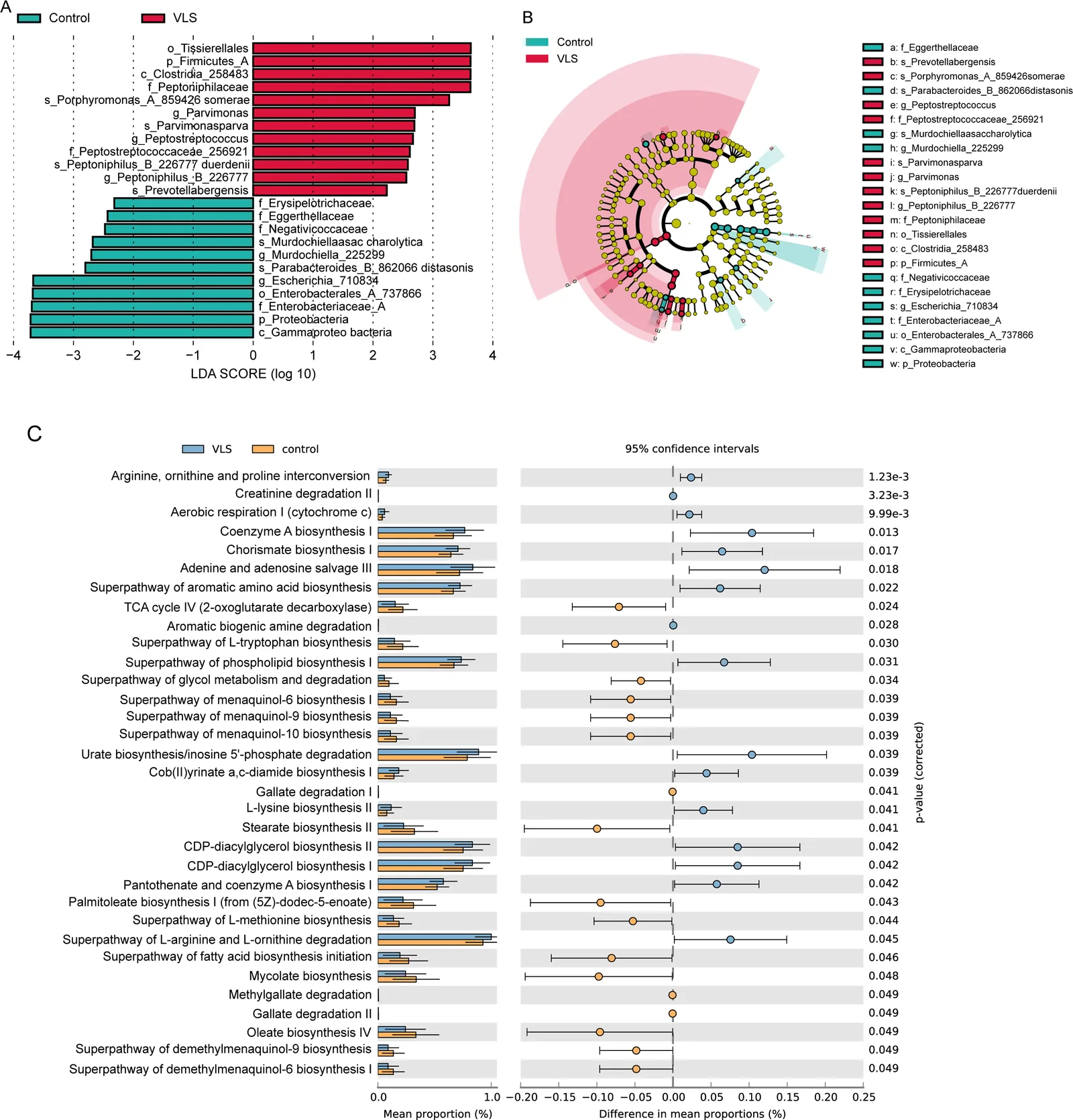
LEfSe and predicted microbial functional pathways in control and VLS groups. (**A**) Histogram of LDA scores for taxa with significant abundance differences (LDA score >2.0, *P* < 0.05). Red bars indicate taxa enriched in VLS (12 taxa, including *o_Tissierellales*, *s_Porphyromonas_A_859426 somerae*, and *g_Parvimonas*), and blue bars indicate taxa enriched in controls (11 taxa, including *p_Proteobacteria* and *g_Escherichia_710834*). (**B**) Taxonomic cladogram showing the phylogenetic distribution of differentially abundant taxa. Each node represents a specific taxonomic type. Red/blue nodes represent taxa more abundant in the VLS/control group, and yellow nodes show no significant differences. (**C**) Comparison of MetaCyc pathways between different bacterial groups was performed, and pathways with significant differences between the two groups were screened out. The left histogram represents the abundance percentage of a metabolic pathway among all metabolic pathways in the two sample sets, while the right histogram shows the corrected *P* value using Benjamini-Hochberg correction.

Linear discriminant analysis effect size (LEfSe) identified 12 taxa that were over-represented in VLS patients and 11 taxa over-represented in controls (Fig. 3A and B). The VLS group was characterized by a higher abundance of taxa including *o_Tissierellales*, *p_Firmicutes_A*, *c_Clostridia_258483*, *f_Peptoniphilaceae*, *s_Porphyromonas_A_859426 somerae*, *g_Parvimonas*, *s_Parvimonasparva*, *g_Peptostreptococcus*, *f_Peptostreptococcaceae_256921*, *s_Peptoniphilus_B_226777 duerdenii*, *g_eptoniphilus_B_226777*, and *s_Prevotellabergensis*. In contrast, the control gut microbiota was dominated by *c_Gammaproteobacteria*, *p_Proteobacteria*, *f_Enterobacteriaceae_A*, *o_Enterobacterales_A_737866*, *g_Escherichia_710834*, *Parabacteroides_B_862066 distasonis*, *g_Murdochiella_225299*, *s_Murdochiella asaccharolytica*, *f_Negativicoccaceae*, *f_Eggerthellacea*, and *f_Erysipelotrichaceae*.

Functional predictions of the gut microbiota using PICRUSt2 identified 33 metabolic pathways that differed significantly between VLS and control groups (Fig. 3C; File S5). The VLS enriched pathways included “arginine, ornithine, and proline interconversion,” “aerobic respiration I,” “superpathway of phospholipid biosynthesis I,” “coenzyme A biosynthesis I,” “chorismate biosynthesis I,” “adenine and adenosine salvage III,” and “superpathway of aromatic amino acid biosynthesis” (Fig. 3C). Conversely, the enriched pathways in the control group included “superpathway of aromatic amino acid biosynthesis,” “superpathway of L-tryptophan biosynthesis,” “superpathway of L-methionine biosynthesis,” “stearate biosynthesis II,” and “palmitoleate biosynthesis I” (Fig. 3C).

### Serum metabolic profiles distinguished VLS from control subjects

**Fig 4:**
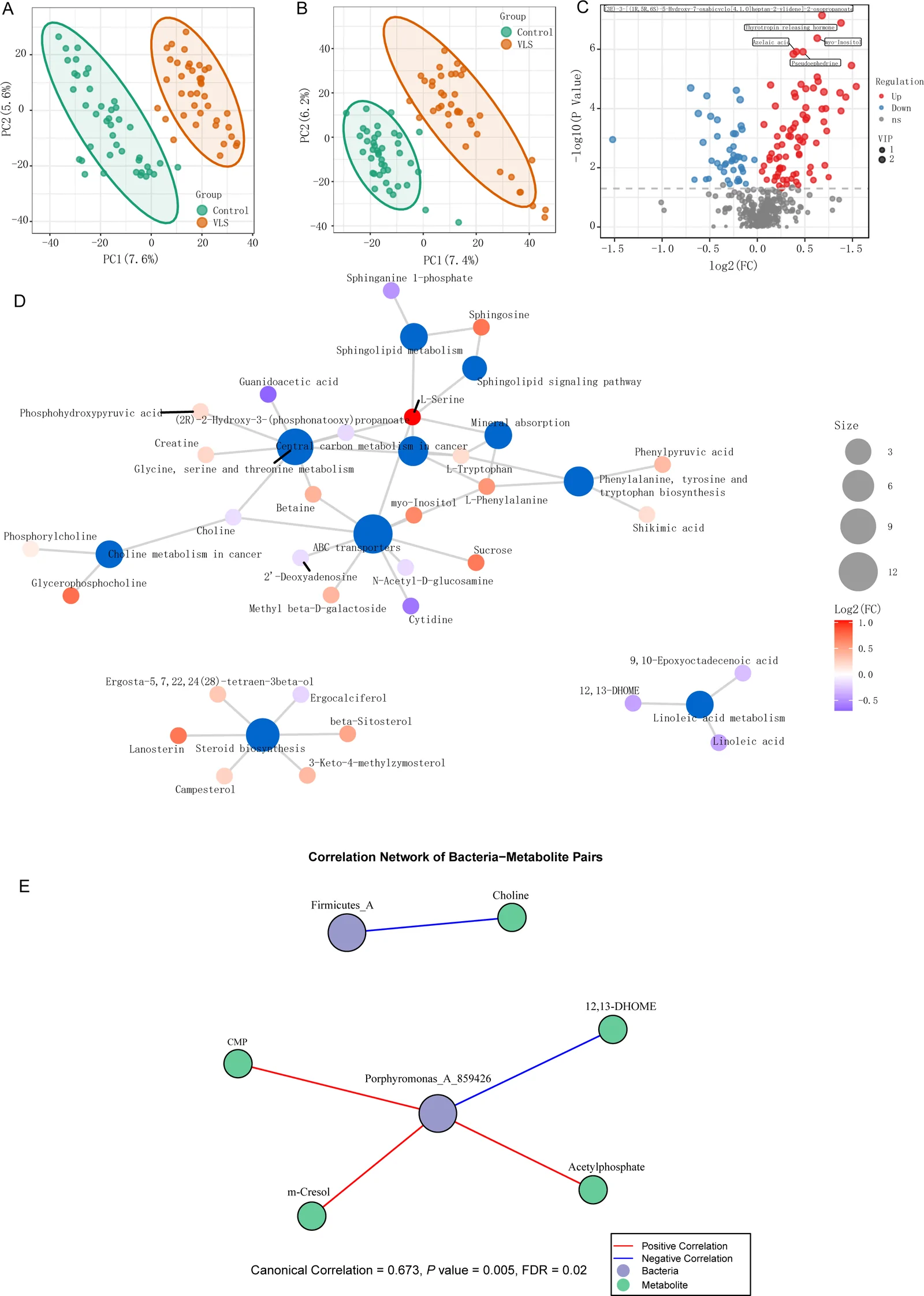
Metabolomic analysis and microbe-metabolite correlations in control and VLS groups. (**A and B**) PLS-DA score plots for positive (**A**) and negative (**B**) ion modes showing separation between VLS and control groups. (**C**) Volcano plot of differentially abundant metabolites between control and VLS groups, with significant metabolites (VIP > 1, *P* < 0.05, fold change > 1.5) highlighted. Among 411 identified metabolites, 79 were up-regulated and 44 were down-regulated in VLS. (**D**) Metabolic pathway network plot, blue nodes represent KEGG pathways, with size proportional to the number of connected metabolites; other nodes denote metabolites, colored by log2(FC) values (red for upregulation, blue for downregulation, with intensity reflecting fold change magnitude). (**E**) Correlation network of top bacteria-metabolite pairs from canonical correlation analysis (CCA) (*k* = 4). Purple nodes: bacteria; green nodes: metabolites; red edges: positive correlations; blue edges: negative correlations. Canonical correlation = 0.673 (*P* = 0.005, false discovery rate [FDR] = 0.02).

Partial least squares discriminant analysis (PLS-DA) illustrated clear clustering and separation between VLS and control groups in both positive and negative ion modes (Fig. 4A and B). PLS-DA showed clear separation between groups, with *Q*^2^ values of 0.891 (positive mode) and 0.873 (negative mode), supporting the reliability of model discrimination despite moderate explained variance of PC1 and PC2, which reflects inherent heterogeneity in clinical samples. To identify the specific metabolites driving this separation, we calculated variable importance in projection (VIP) scores from the orthogonal partial least squares discriminant analysis (OPLS-DA) models. Metabolites with VIP > 1, *P* < 0.05, and fold change > 1.5 were considered key contributors to the group discrimination. In total, 411 metabolites were identified, including 287 in positive mode and 124 in negative mode (Fig. 4C; File S6). Compared with controls, 79 metabolites were up-regulated, and 44 metabolites were down-regulated in the VLS group. The separation along the PLS-DA axes was primarily driven by up-regulated metabolites in VLS such as thyrotropin-releasing hormone, myo-inositol, azelaic acid, phenylpyruvic acid, L-serine, rosmarinic acid, and L-sorbose, as well as down-regulated metabolites including deoxycorticosterone acetate and guanidoacetic acid (Fig. 4C; File S7).

Pathway analysis using MetaboAnalyst revealed significant perturbations in several metabolic pathways (Fig. 4D;File S8). The most notably altered pathways in VLS included phenylalanine, central carbon metabolism in cancer, linoleic acid metabolism, mineral absorption, sphingolipid signaling pathway, and sphingolipid metabolism.

### Overlap analysis between predicted microbial functions and observed metabolic pathways

By aligning functional pathway annotations from 16S rRNA sequencing (Fig. 3C) and metabolomics (Fig. 4D), we observed overlapped pathways in amino acid metabolism, lipid metabolism, and energy metabolism (Table 2). In amino acid metabolism, both data sets converged on pathways related to aromatic amino acid biosynthesis (e.g., “phenylalanine, tyrosine, and tryptophan biosynthesis” from metabolomics versus “superpathway of aromatic amino acid biosynthesis” from 16S). For lipid metabolism, shared pathways included unsaturated fatty acid metabolism (e.g., “linoleic acid metabolism” from metabolomics versus “stearate biosynthesis II” and “palmitoleate biosynthesis I” from 16S). Regarding energy metabolism, both approaches consistently implicated alterations in the tricarboxylic acid (TCA) cycle and related energy-generating processes. Specifically, “Central carbon metabolism in cancer” identified from metabolomics, which includes glycolysis and TCA crosstalk, aligned with 16S pathways such as “TCA cycle IV” and “aerobic respiration I.” These convergences highlight the coordinated perturbations in core metabolic processes at both the gut microbiota and the host metabolic levels, providing a systemic view of their functional interplay.

**TABLE 2:** Overlapped pathways between 16S rRNA sequencing and metabolomics

Common pathway category	Pathway name in metabolomics	Pathway name in 16S	Metabolomics *P* value	16S *P* value
Amino acid metabolism	Phenylalanine, tyrosine, and tryptophan biosynthesis	Superpathway of aromatic amino acid biosynthesis	0.0217	0.0318
Lipid metabolism	Linoleic acid metabolism	Stearate biosynthesis II, palmitoleate biosynthesis I	0.0416	0.0409, 0.0430
Energy metabolism	Central carbon metabolism in cancer (involving glycolysis/TCA cycle)	TCA cycle IV, aerobic respiration I (dependent on TCA cycle)	0.0380	0.0239, 0.0099

### Integrated analysis revealed convergence of microbial and metabolic alterations

CCA was performed using the differentially abundant bacterial genera and serum metabolites to explore interactions between the gut microbiota and host metabolism. This analysis revealed a significant multivariate relationship with a canonical correlation coefficient of 0.673 (*P* = 0.005, FDR = 0.02; Fig. 4E), identifying specific bacterium-metabolite pairs. Notably, *s_Porphyromonas_A_859426_somerae* exhibited positive correlations with acetylphosphate, CMP, and m-cresol and a negative correlation with 12,13-DHOME, highlighting its potential role in modulating host metabolic pathways.

### Microbial and metabolic biomarkers showed high diagnostic potential

**Fig 5:**
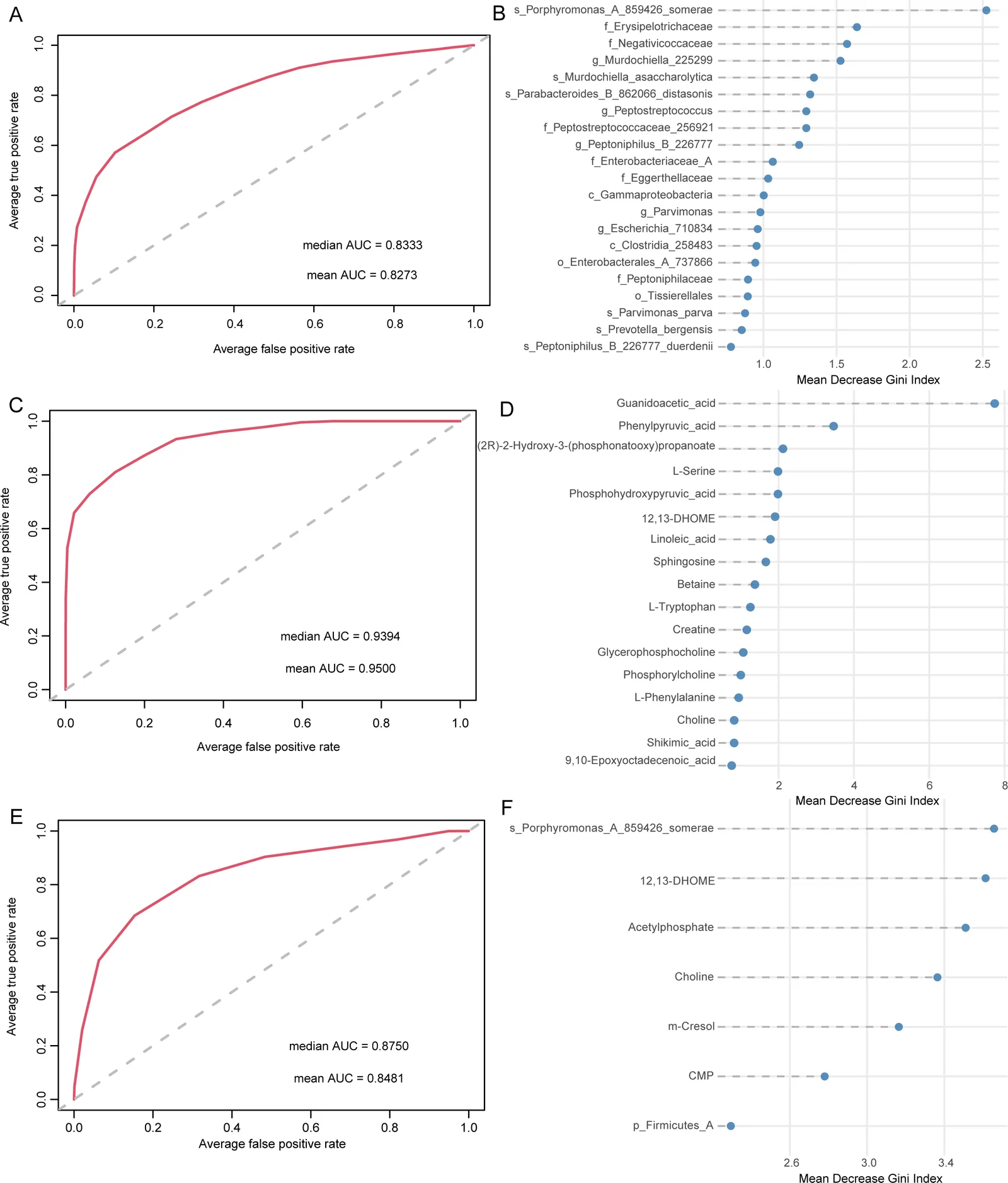
Diagnostic performance of microbial, metabolic, and integrated biomarkers. (**A, C, and E**) ROC curves for the microbial taxa model (**A**), the differential metabolite model (**C**), and the integrated model combining two bacterial taxa and five metabolites (**E**). The median and mean AUC values are displayed on each plot. (**B, D, and F**) Mean Decrease Gini Index plots showing the top contributing features for the microbial model (**B**), the metabolic model (**D**), and the integrated model (**F**). The *x*-axis represents the mean decrease in Gini index, and the *y*-axis lists the features.

The diagnostic performance of microbial and metabolic biomarkers was evaluated using receiver operating characteristic (ROC) analysis. Microbial taxa identified by LEfSe achieved an area under the curve (AUC) of 0.83, indicating moderate accuracy in distinguishing VLS from controls (Fig. 5A). Mean Decrease Gini Index analysis highlighted key taxa, particularly *s_Porphyromonas_A_859426_somerae*, as the primary contributor to classification (Fig. 5B). The metabolic biomarker model demonstrated higher discriminatory power, with an AUC of 0.93 (Fig. 5C), and guanidinoacetic acid was identified as a top feature (Fig. 5D). Although other metabolites showed larger fold changes, guanidinoacetic acid was identified as the top feature by the random forest model based on its high mean decrease Gini index, indicating its strong contribution to classification accuracy when combined with other metabolites. Finally, an integrated model combining both the top two microbial features and five differential metabolites maintained robust performance at an AUC of 0.87 (Fig. 5E), with *s_Porphyromonas_A_859426_somerae*, 12,13-DHOME, and acetylphosphate serving as principal contributors (Fig. 5F). These results demonstrate the complementary value of multi-omics biomarkers in VLS identification.

## DISCUSSION

The study integrates gut microbiome and serum metabolome analyses to explore potential associations related to the gut-skin axis in prepubertal VLS. Our principal finding is that overall microbial diversity and community structure were preserved, while modest differences in specific gut bacterial taxa and systemic metabolic profiles were observed. These features showed exploratory classification potential within this data set. These results suggest that subtle variations in the gut microbiome composition and host metabolism may be present in prepubertal VLS, even before the large-scale shifts in microbial community structure.

In contrast to the diversity differences reported in five prepubertal VLS cases by Chattopadhyay et al. (10), we did not observe significant alpha diversity differences in our cohort. This discrepancy may be attributable to differences in sample size, ethnicity, diet patterns, or disease duration. Similarly, studies in perimenopausal or postmenopausal women with VLS have reported reduced α diversity (13), potentially reflecting the cumulative effects of aging, prolonged disease chronicity, and age-related decline in immune regulation and gut barrier function (14–16). The preserved microbial diversity in our cohort suggests that relative structural stability of the microbiome in prepubertal VLS, while specific functional and metabolic disturbances are already present. This observation may be associated with the early disease stage and age-related host factors, such as differences in immune regulation, although further studies are needed to confirm these mechanisms.

At the taxonomic level, our observations both align and contrast with prior work. Chattopadhyay et al. reported increased abundances of both *Escherichia* and *Dialister* in VLS patients (10). In our cohort, *Dialister* was non-significantly increased, whereas *Escherichia_710834* showed a trend toward decreased abundance. These discrepancies may reflect differences in cohort characteristics or sample size across studies. Given the limited taxonomic resolution of 16S rRNA sequencing, the functional implications of these genus-level differences remain unclear. Similarly, although pro-inflammatory taxa such as *Peptoniphilus* were over-represented in VLS, their potential roles should be interpreted with caution, as no direct functional or mechanistic evidence is provided in the current study.

Beyond taxonomic shifts, our functional and metabolic findings revealed coherent alterations in host-microbe crosstalk. PICRUSt2 analysis predicted enrichment in microbial pathways involved in arginine/proline metabolism and aerobic respiration in VLS. Serum metabolomics confirmed and expanded these findings, highlighting disruptions in glycine/serine/threonine metabolism, sphingolipid metabolism, and linoleic acid metabolism. The high concordance between predicted microbial functions and observed host metabolic perturbations may reflect a potential association between functional shifts in the gut microbiota and systemic metabolic disturbances in VLS, potentially affecting immune modulation, epithelial integrity, and inflammation (17).

Integrated analysis provided additional mechanistic insight into host-microbe associations. CCA revealed significant correlations between the *Porphyromonas_A_859426* and metabolites, including 12,13-DHOME and acetylphosphate. *Porphyromonas* species are anaerobic bacteria that produce proteases and lipopolysaccharides that have been associated with inflammatory responses and tissue remodeling (18). 12,13-DHOME, known as isoleukotoxin diol, is an oxylipin derived from linoleic acid metabolism and has been implicated in pain perception and immune regulation, potentially promoting persistent inflammation through T-cell modulation (19, 20). A recent single-cell and spatial transcriptomics analysis revealed that reduction of immune regulation may facilitate the reactivation of tissue-resident memory T cells in vulvar skin, which, together with keratinocytes, fibroblasts, and melanocytes, amplifies local inflammation and tissue damage (21). The correlation suggests a coordinated host-microbe metabolic network in which altered microbial composition and microbial-derived metabolites may synergistically influence host inflammatory and metabolic pathways, potentially contributing to VLS pathophysiology.

The classification models based on microbial and metabolic features showed good performance within this data set. Serum metabolites alone achieved high accuracy, suggesting that metabolic alterations may serve as sensitive indicators of the host’s disease state. The integrated model combining microbial and metabolic features maintains robust performance, reflecting the complementary value of microbiome-associated and host metabolic signatures in characterizing VLS-associated alterations.

Several limitations should be acknowledged. First, the cross-sectional design limits the ability to draw causal inferences. Second, 16S rRNA sequencing restricts taxonomic resolution and precludes strain-level functional analyses. Third, functional profiling of the gut microbiota relied on inference from PICRUSt2 rather than direct metagenomics. Thus, the proposed links between microbial functions and host metabolism require validation through direct functional metagenomic approaches. Future longitudinal, multicenter studies integrating metagenomics, targeted metabolomics, and mechanistic experiments are needed to establish causality and clarify the gut-microbes to vulvar inflammation. Fourth, we did not collect detailed dietary or lifestyle metadata for study participants. Although all subjects were recruited from the same geographic region and were age-matched to reduce potential heterogeneity, and recent antibiotic, probiotic, or anti-inflammatory medication use as well as preexisting autoimmune and metabolic diseases were excluded, unaccounted confounding effects from diet and lifestyle cannot be fully ruled out. Future investigations should collect and adjust for comprehensive dietary, lifestyle, and clinical metadata to further validate the observed associations.

In conclusion, our study identifies modest differences in gut microbial composition and serum metabolic profiles in prepubertal VLS, along with associations between specific microbes and host metabolites. These findings provide preliminary evidence of interactions between the gut microbiome and host metabolism in this condition and suggest that integrated microbial and metabolic signatures may serve as exploratory non-invasive biomarkers for VLS detection, particularly in pediatric populations in whom invasive procedures are limited. Understanding these gut-skin interactions may also provide a basis for future strategies aimed at restoring microbial and metabolic homeostasis in VLS. However, larger, longitudinal cohorts, together with functional studies, are required to validate these observations and clarify their biological and clinical relevance.

## MATERIALS AND METHODS

### Sample recruitment

A total of 89 participants were enrolled in the study, comprising 43 prepubertal girls with VLS and 46 healthy controls recruited from the Children’s Hospital, Zhejiang University School of Medicine. All participants had not received antibiotics, probiotics, or anti-inflammatory medications in the preceding 6 months, and none had known autoimmune or metabolic diseases. Healthy controls were recruited from the same hospital from children undergoing external genital nevus removal or redundant hymen surgery. Healthy controls were recruited from the same hospital during routine physical checkups. They had no history of anogenital diseases, autoimmune diseases, or any chronic inflammatory conditions. Fecal samples were successfully obtained from 75 individuals (42 VLS patients and 33 controls) using sterile cotton swabs inserted 2–3 cm into the anal canal under aseptic conditions. Samples were immediately stored at −80°C until DNA extraction.

Peripheral blood samples were collected from 81 participants (39 VLS patients and 42 controls). Among them, 67 individuals (38 VLS patients and 29 controls) provided paired fecal and blood samples for paired comparative analysis. Blood samples were centrifuged at 3,000 rpm for 10 min to separate serum, which was then transferred to sterile Eppendorf tubes and stored at −80°C for downstream analysis.

### Fecal DNA extraction and sequencing of the 16S rRNA gene

Microbial genomic DNA was extracted from fecal samples using the OMEGA Soil DNA Kit (M5635-02) (Omega Bio-Tek, Norcross, GA, USA). The hypervariable V3–V4 regions of bacterial 16S rRNA genes were amplified with barcoded specific primers 338F (5′-ACTCCTACGGGAGGCAGCAG-3′) and 806R (5′-GGACTACHVGGGTWTCTAAT-3′) using TransStart FastPfu DNA Polymerase (TransGen Biotech, China) on an ABI GeneAmp 9700 PCR System (Applied Biosystems, USA). PCR products were purified using the AxyPrep DNA Gel Extraction Kit (Axygen Biosciences, USA), quantified with the QuantiFluor-ST Fluorescence Quantification System (Promega, USA), and libraries were prepared with the TruSeq DNA Library Prep Kit (Illumina, USA). Paired-end sequencing (2 × 250 bp) was carried out on an Illumina MiSeq platform (Illumina, USA).

### 16S rRNA data processing

Raw 16S rRNA sequencing reads were initially filtered and assembled using VSEARCH (22) to eliminate low-quality and short-length reads. Subsequent data processing was conducted using QIIME2 (23). Denoising was performed with the DADA2 plugin (24) in QIIME2, which resolves ASVs with single-nucleotide resolution, offering a higher-resolution alternative to traditional 97% similarity-based operational taxonomic unit clustering. Taxonomic classification of ASVs was carried out using the Greengenes2 database (25).

Alpha diversity metrics, including the Shannon index, Evenness, and Faith PD, were calculated to assess within-sample diversity. For alpha diversity analysis, samples were rarefied to a uniform depth of 15,000 reads per sample, which was the minimum sequencing depth across all samples after quality filtering. Beta diversity was evaluated using Bray-Curtis dissimilarity. PCoA was visualized using the vegan package in R.

### Taxonomic biomarker identification

Differences in microbial taxa abundance between VLS and control groups at the phylum and genus levels were assessed using the Wilcoxon rank-sum test, with *P* values adjusted for multiple comparisons using the BH method. To account for potential confounding effects of age and body weight, we further performed multivariable linear regression analyses on log-transformed relative abundances of each taxon, including age and body weight as covariates; the resulting *P* values were again corrected for multiple testing using the BH procedure. LEfSe (https://huttenhower.sph.harvard.edu/lefse/), which combines statistical significance with biological relevance by coupling non-parametric tests with linear discriminant analysis (LDA) (26), was applied to identify significant differential abundance, using an LDA score threshold of 2.0.

### Functional pathway prediction of microbiome

Microbial functional pathways were predicted from 16S rRNA sequencing data using PICRUSt2 based on the MetaCyc database (27, 28). Differences in pathways between the VLS and control groups were determined using STAMP (29) using Welch’s *t*-test with 95% confidence intervals, followed by Benjamini-Hochberg correction for multiple comparisons. Corrected *P* values <0.05 were considered statistically significant.

### Metabolite profiling

Serum samples were prepared as follows: after thawing on ice, each sample was vortexed for 1 min, and an appropriate volume (≤2 mL) was transferred to a 2 mL centrifuge tube. Four hundred microliters of methanol was added, vortexed for 1 min, and centrifuged at 12,000 rpm for 10 min at 4°C. The entire supernatant was collected, concentrated to dryness, and reconstituted in 150 μL of 80% methanol/water containing 2-chlorophenylalanine (4 ppm, internal standard). The reconstituted solution was filtered through a 0.22 μm PTFE membrane prior to LC-MS analysis.

Chromatographic separation was performed on a Waters ACQUITY UPLC System equipped with an ACQUITY UPLC HSS T3 column (2.1 × 100 mm, 1.8 μm) at 40°C. The flow rate was 0.3 mL/min, and the injection volume was 2 μL. For positive ion mode, mobile phases consisted of (A1) 0.1% formic acid in water and (B1) 0.1% formic acid in acetonitrile, using a gradient program: 0–1 min, 8% B1; 1–8 min, 8%–98% B1; 8–10 min, 98% B1; 10–10.1 min, 98%–8% B1; 10.1–12 min, 8% B1. For negative ion mode, mobile phases were (A2) 5 mM ammonium formate in water and (B2) acetonitrile with the same gradient profile.

Mass spectrometry (MS) was performed on a Thermo Q Exactive Orbitrap mass spectrometer equipped with an electrospray ionization source. Full MS scans were acquired over *m*/*z* 100–1,000 at a resolution of 70,000. Data-dependent MS/MS spectra were collected for the top three ions per cycle at a resolution of 17,500 with a normalized collision energy of 30 eV. Dynamic exclusion was used to reduce redundant MS/MS scans.

All study samples were analyzed in a single batch without interruption. A pooled QC sample was prepared by mixing equal aliquots of all study samples and was injected every 10 injections to monitor system stability. Because the entire run was completed in one batch, no additional batch effect correction was required; the frequent QC injections confirmed that retention time shifts and signal intensity drifts were negligible throughout the analysis.

### Metabolomic data analysis

Raw MS data were converted to mzXML format using MSConvert from the ProteoWizard software suite (30). Feature detection, retention time alignment, and peak picking were performed in R using the XCMS package (31) with the centWave algorithm (mass tolerance ppm = 15, peak width range 5–30 s, and a minimum *m*/*z* difference 0.01). QC samples were used to correct for batch effects. Metabolites with a relative standard deviation > 30% in QC samples were excluded from downstream analysis. Metabolites were identified using high-resolution mass spectrometry and tandem MS (MS/MS) data by matching accurate mass, retention time, and MS/MS fragmentation patterns against multiple public databases, including HMDB, MassBank, KEGG, LipidMaps, and mzCloud (32–36), as well as an in-house database from Panomix Biomedical Tech Co., Ltd. (Suzhou, China).

Multivariate analysis was performed using the ropls R package (37). Both unsupervised principal component analysis and supervised PLS-DA with orthogonal signal correction (OPLS-DA) were used to assess group separation and identify discriminant metabolites. Model quality was evaluated using the predictive ability *Q*^2^ from 10-fold cross-validation, with *Q*^2^ > 0.5 considered indicative of a robust and predictive model. Metabolites contributing to group separation were identified using VIP scores. Differential metabolites were defined based on *P* value <0.05 from a two-tailed Student’s *t*-test (corrected by the Benjamini-Hochberg correction), VIP > 1 from the OPLS-DA model, and fold change values >1.5 between groups.

### Pathway enrichment and visualization

Differential metabolites were subjected to pathway enrichment and topology analysis using MetaboAnalyst (38). The identified metabolites were mapped to KEGG pathways for functional interpretation, with results visualized via the KEGG Mapper (39) to highlight perturbed metabolic networks.

### Integrated analysis of metabolomics and 16S rRNA sequencing data

To explore potential interactions between gut microbiota and host metabolism, an integrated analysis was performed by correlating 16S rRNA sequencing data with metabolomic data. Taxa identified as differentially abundant through LEfSe were correlated with significantly altered metabolites. CCA was performed using the PMA R package, with the number of canonical components set to 4 (*k* = 4) based on scree plot evaluation and model performance. Statistical significance was assessed via 1,000 permutations, with *P* values adjusted using the FDR method.

### Random forest-based biomarker evaluation

To evaluate the diagnostic potential of microbial biomarkers, a random forest classification model was implemented in R using the randomForest package. The model was trained on the identified discriminant taxa with 100 iterations to mitigate overfitting. Model performance was evaluated by ROC analysis using the ROCR package. The ROC curve was generated by plotting the true positive rate (sensitivity) against the false positive rate (1−specificity), with AUC values ≥0.7 considered indicative of robust discriminatory power.

## Supplementary Material

Reviewer comments

## Data Availability

The raw 16S sequence data reported in this paper have been deposited in the Genome Sequence Archive (GSA; for a description of the GSA database, see Genomics, Proteomics & Bioinformatics 2025) in National Genomics Data Center (NGDC; for a description of NGDC, see Nucleic Acids Res 2025), China National Center for Bioinformation/Beijing Institute of Genomics, Chinese Academy of Sciences (GSA: CRA034514) that are publicly accessible at https://ngdc.cncb.ac.cn/gsa. The metabolomics data reported in this paper have been deposited in the OMIX, China National Center for Bioinformation/Beijing Institute of Genomics, Chinese Academy of Sciences (https://ngdc.cncb.ac.cn/omix: accession no. OMIX016393).

## SUPPLEMENTAL MATERIAL

Figure S1 spectrum.04058-25-s0001.tif Rarefaction curves of gut microbiota in control and VLS groups.

File S1 spectrum.04058-25-s0002.docx Sample metadata for 16S rRNA sequencing and metabolomics analysis.

File S2 spectrum.04058-25-s0003.docx Demographic and clinical characteristics of the study population.

File S3 spectrum.04058-25-s0004.docx Relative abundance of microbial communities at phylum.

File S4 spectrum.04058-25-s0005.docx Functional alterations of metabolic pathways compared VLS with control subjects.

File S5 spectrum.04058-25-s0006.docx Metabolites identified in positive and negative mode.

File S6 spectrum.04058-25-s0007.docx Differential metabolites in VLS patients compared with the control group.

File S7 spectrum.04058-25-s0008.docx Pathways related to the differential metabolites screened by 586 MetaboAnalyst.

File S8 spectrum.04058-25-s0009.docx Miscellaneous pathways related to the differential metabolites screened by MetaboAnalyst.

Figure S1 Legend spectrum.04058-25-s0010.docx Rarefaction curves of gut microbiota in control and VLS groups.

OPEN PEER REVIEW reviewer-comments.pdf An accounting of the reviewer comments and feedback.

ASM does not own the copyrights to Supplemental Material that may be linked to, or accessed through, an article. The authors have granted ASM a non-exclusive, world-wide license to publish the Supplemental Material files. Please contact the corresponding author directly for reuse.
