# Supplementary material for: Integrated microbiome and metabolome profiling in prepubertal girls with vulvar lichen sclerosus
Source: Microbiol Spectr. 2026 Aug 27;14(10):e04058-25. doi: 10.1128/spectrum.04058-25 (PMC13689091; doi:10.1128/spectrum.04058-25)
Supplement: Supplementary file 1 [file reviewer-comments.pdf]

AMERICAN SOCIETY FOR MICROBIOLOGY | Microbiology Spectrum

Integrated Microbiome and Metabolome profiling in Prepubertal Girls with Vulvar Lichen Sclerosus

Huihui Gao, Fengxia Li, Hongyu Chen, Siming Wu, Junwen Zhang, Chaoqun Wang, Qiuxiang Shen, Zhu Li, Dandan Chen, Lingling Jin, Jie Liu, Jiayu Shen, Liying Sun, and Lan YU

Corresponding Author(s): Lan YU, Zhejiang University

Review Timeline:

Submission Date:	January 13, 2026
Editorial Decision:	April 12, 2026
Revision Received:	May 18, 2026
Accepted:	June 9, 2026

Editor: Xinzhao Tong

Reviewer(s): The reviewers have opted to remain anonymous.

Transaction Report:

(Note: With the exception of the correction of typographical or spelling errors that could be a source of ambiguity, letters and reports are not edited. The original formatting of letters and referee reports may not be reflected in this compilation.)

DOI: https://doi.org/10.1128/spectrum.04058-25

Re: Spectrum04058-25 (**Integrated Microbiome-Metabolome Analysis Reveals Pathogenic Mechanisms and Biomarkers in Prepubertal Girls with Vulvar Lichen Sclerosus**)

Dear Dr. Lan YU:

Thank you for the privilege of reviewing your work. Below you will find my comments, instructions from the Spectrum editorial office, and the reviewer comments.

The reviewers' major concerns are overinterpretation of the results and statistical issues in the data analysis, both of which need careful attention. In addition, the deposited data needs to be released to the public.

Please return the manuscript within 60 days; if you cannot complete the modification within this time period, please contact me. If you do not wish to modify the manuscript and prefer to submit it to another journal, notify me immediately so that the manuscript may be formally withdrawn from consideration by Spectrum.

Revision Guidelines
To submit your modified manuscript, log into the submission site at https://spectrum.msubmit.net/cgi-bin/main.plex. Go to Author Tasks and click the appropriate manuscript title to begin. The information you entered when you first submitted the paper will be displayed; update this as necessary. Note the following requirements:

• Upload point-by-point responses to the issues raised by the reviewers in a file named "Response to Reviewers," NOT in your cover letter.
• Upload a compare copy of the manuscript (without figures) as a "Marked-Up Manuscript" file.
• Upload a clean .DOC/.DOCX version of the revised manuscript and remove the previous version.
• Each figure must be uploaded as a separate, editable, high-resolution file (TIFF or EPS preferred), and any multipanel figures must be assembled into one file.
• Any supplemental material intended for posting by ASM should be uploaded with their legends separate from the main manuscript. You can combine all supplemental material into one file (preferred) or split it into a maximum of 10 files with all associated legends included.

For complete guidelines on revision requirements, see our Submission and Review Process webpage. Submission of a paper that does not conform to guidelines may delay acceptance of your manuscript.

Data availability: ASM policy requires that data be available to the public upon online posting of the article, so please verify all links to sequence records, if present, and make sure that each number retrieves the full record of the data. If a new accession number is not linked or a link is broken, provide Spectrum production staff with the correct URL for the record. If the accession numbers for new data are not publicly accessible before the expected online posting of the article, publication may be delayed; please contact production staff (Spectrum@asmusa.org) immediately with the expected release date.

Publication Fees: For information on publication fees and which article types are subject to charges, visit ourwebsite. If your manuscript is accepted for publication and any fees apply, you will be contacted separately about payment during the production process; please follow the instructions in that e-mail. Arrangements for payment must be made before your article is published.

ASM Membership: Corresponding authors may join or renew ASM membership to obtain discounts on publication fees. Need to upgrade your membership level? Please contact Customer Service at Service@asmusa.org.

The ASM Journals program strives for constant improvement in our submission and publication process. Please tell us how we can improve your experience by taking this quick Author Survey.

Thank you for submitting your paper to Spectrum.

Sincerely,
Xinzhao Tong
Editor
Microbiology Spectrum

Reviewer #1 (Comments for the Author):

Vulvar lichen sclerosus (VLS) is a chronic, autoimmune skin condition characterized by white patches in the anogenital region and an increased risk of squamous cell cancer. Although it primarily affects post-menopausal women, it also affects prepubertal girls at a lower frequency. Gut dysbiosis has been reported in smaller, earlier studies. The article by Gao and colleagues uses a

combination of 16S rRNA marker gene-based gut microbiome profiling and serum metabolomics to assess changes in prepubertal girls suffering from vulvar lichen sclerosis, relative to a group of age-matched controls. The study aims to identify gut microbial taxa and metabolites with diagnostic potential. Microbiome profiling, followed by alpha- and beta-diversity analyses, did not reveal significant differences in these parameters between the control and VLS groups. Differential abundance testing, however, showed that Escherichia_710834 showed a very small but significant decrease in abundance in VLS samples compared to controls. Altered microbial metabolic function potential was predicted from the taxa abundance data (16S rRNA gene sequencing). The authors show that some aromatic amino acid synthesis, fatty acid biosynthesis and aerobic respiration-related pathways were predicted to be differentially abundant. Metabolome analysis appears to distinguish between the control and VLS group, as shown in the PLS-DA analysis. The authors then identify microbial taxa and metabolites that may be of diagnostic utility. The study asks a relevant question, and the data do not appear to show strong differences between the VLS and control groups.

Concerns:
1. The title of the manuscript needs to be revised as the study neither explains pathogenic mechanisms nor identifies valid biomarkers for VLS, which have been tested.
2. The major concern with the manuscript is that minor variations in the data appear to be overinterpreted.
3. Several factors, including the diet, lifestyle, non-antibiotic medication, supplements etc., can contribute to individual variability in the gut microbiota. Have the authors recorded such metadata and adjusted for variations in these before interpreting the findings?
4. The PCoA plot for 16S rRNA gene sequencing data may be modified to indicate what percentage of the variation is explained by each axis.
5. In the metabolome profiling experiment, the PLS-DA analysis plot shows that very less variation in the data is explained by the PC1 (~7) and PC2 (~6) axes.
6. How do the authors explain the separation along these axes? Have VIP scores been calculated to identify metabolites driving the separation between the groups?
7. The figure legends merely describe the figures, but do not convey the main findings/interpretations from the figures. Inclusion of detailed legends will make figures self-explanatory.
8. Access to the data generated in the study is restricted by a login step.
In summary, there appear to be minor differences in the microbiome and metabolite profiles between the VLS and control groups in the age group of affected individuals. It may be useful to modify the manuscript accordingly.

Reviewer #2 (Comments for the Author):

In this manuscript, the authors performed 16S sequencing (feces) and untargeted metabolomics (serum) on a cohort of approx. 90 prepubertal girls with vulvar lichen sclerosus. The study is well designed and cohort size is sufficient. The authors analyzed both microbiome and metabolomics data separately, as well as integreated both data types. The statistical analysis seems appropriate, the manuscript is well written and the results are interesting, and I believe both the data and the analysis will be of interest to the field. There are some comments that I think should be addressed to improve the manuscript:

General comments:
I think adding a table summarizing the two groups (healthy controls and prepubertal VLS) and detailing basic demographics would be helpful. It would be good to include the number of samples with 16S and metabolomics, age range, and any other relevant information, including statistics on comparison of the different demographics (such as age) between the groups.
16S rRNA data processing: were samples rarified prior to alpha diversity analysis? if so, to what depth (please specify in the methods)? If not, rarefaction needs to be applied prior to analysis.

Results:
L110: is it 33 healthy controls as stated here? or 45 healthy controls as stated in the abstract (line 48)? also 44 prepubertal VLS (as in the abstract) or 42? I see it is explained in the methods (that some samples had only 16S or only metabolomics), but would be good to clarify this in the results as well.

L124: " decreased Proteobacteria in VLS ($P = 0.041$, wilcoxon test)" - can you also provide the FDR corrected q-value?

Table1 / Table2 - "P values were calculated via the Wilcoxon rank-sum test, with significance set at $P < 0.05$. Benjamini-Hochberg (BH) correction was used for multiple-testing adjustment." Are the values listed in the table the raw p-values or the FDR corrected q-values? Please clarify in the table legend (and if they are the raw p-values, please also provide the FDR corrected q-values in the table as well).

L200: "Guanidinoacetic acid was identified as a top feature" - It seems there are a lot of metabolites that show a stronger difference between the groups comapred to guanidinoacetic acid (both in fold change and p-value). Do you have any speculations as to why this metabolite was the top feature?

Methods:
L379: "Sample recruitment" - how were the controls recruited? Were they healthy volunteers? Or children with other conditions?

L393: "written informed consent was obtained from each participant" - since the participants are children, was consent obtained from their parents or legal guardians? Please clarify.

L424: Functional pathway prediction of microbiome - does not state FDR correction for multiple testing, whereas Fig3C the p-value (and in the legend) says "corrected". What correction is it?

L444: "Differential metabolites were defined based on P-value < 0.05" - How were the p-values calculated? Was there any correction for multiple testing? If so, please specify the method used and provide the corrected p-values (q-values) in the results as well.

L463: "identified discriminant taxa with 100-fold cross-validation" - how was 100-fold cross-validation performed if only less than 100 samples were present?

Please include in the methods section some more experimental details about the mass-spectrometry (type of mass-spectrometer used, sample preparation, number of ms2 spectra (was it on each sample? on pooled samples?) etc.). Also, were all metabolomics samples run in a single batch? If not, were samples randomized into batches? How were batch effects controlled for?

Data availability:
L289: "The raw sequence data reported in this paper have been deposited in the Genome Sequence Archive (Genomics, Proteomics & Bioinformatics 2025) in National Genomics Data Center (Nucleic Acids Res 2025)" - are "(Genomics, Proteomics & Bioinformatics 2025)" and "(Nucleic Acids Res 2025)" references? to what?

Could the authors also provide access to the metabolomics data (raw and/or processed) in a public repository? This would allow other researchers to validate the findings and perform further analyses.

Minor
L47: "its"->"the"
Supplemental figure S1B: Is it after rarefaction? Also, why are there negative values in the violin plot? Also, I do not see any samples in FigS1A with < 150ASVs, but in FigS1B it seems there are samples with < 150ASVs (mainly in the control group)? Also, what is the difference between FigS1B and Figure 1E? Do they show the same thing? If so, why are they different? If not, please clarify.

Reviewer #1 (Comments for the Author):

Vulvar lichen sclerosus (VLS) is a chronic, autoimmune skin condition characterized by white patches in the anogenital region and an increased risk of squamous cell cancer. Although it primarily affects post-menopausal women, it also affects prepubertal girls at a lower frequency. Gut dysbiosis has been reported in smaller, earlier studies. The article by Gao and colleagues uses a combination of 16S rRNA marker gene-based gut microbiome profiling and serum metabolomics to assess changes in prepubertal girls suffering from vulvar lichen sclerosis, relative to a group of age-matched controls. The study aims to identify gut microbial taxa and metabolites with diagnostic potential. Microbiome profiling, followed by alpha- and beta-diversity analyses, did not reveal significant differences in these parameters between the control and VLS groups. Differential abundance testing, however, showed that Escherichia_710834 showed a very small but significant decrease in abundance in VLS samples compared to controls. Altered microbial metabolic function potential was predicted from the taxa abundance data (16S rRNA gene sequencing). The authors show that some aromatic amino acid synthesis, fatty acid biosynthesis and aerobic respiration-related pathways were predicted to be differentially abundant. Metabolome analysis appears to distinguish between the control and VLS group, as shown in the PLS-DA analysis. The authors then identify microbial taxa and metabolites that may be of diagnostic utility. The study asks a relevant question, and the data do not appear to show strong differences between the VLS and control groups.

Concerns:

1. The title of the manuscript needs to be revised as the study neither explains pathogenic mechanisms nor identifies valid biomarkers for VLS, which have been tested.

Response: We sincerely appreciate this valuable suggestion. We have revised the title to a more cautious and accurate version:

“Integrated Microbiome and Metabolome profiling in Prepubertal Girls with Vulvar

Lichen Sclerosus”

2. The major concern with the manuscript is that minor variations in the data appear to be overinterpreted.

Response: We sincerely appreciate this critical and constructive comment. We agree that some of the initial interpretations related to microbial and metabolic differences were overinterpreted. In the revised manuscript, we have carefully moderated the language throughout the Results and Discussion sections to ensure that our conclusions more accurately reflect the exploratory nature of the study and the modest effect sizes observed (Line 51, page 3; Line 65-68, page 3; Line 81-83, page 4; Line 115-116, page 6; Line 238-239, page 11; Line 308-309, page 15; Line 310-313, page 15).

Specifically, we have revised statements implying causality or mechanistic inference and replaced them with more conservative wording emphasizing associations and potential relationships. We have also reduced overinterpretation of biomarker potential by consistently referring to these findings as exploratory and preliminary.

We believe these revisions have improved the balance between data interpretation and the level of evidence provided, and better align the conclusions with the scope of the study.

3. Several factors, including the diet, lifestyle, non-antibiotic medication, supplements etc., can contribute to individual variability in the gut microbiota. Have the authors recorded such metadata and adjusted for variations in these before interpreting the findings?

Response: We thank the reviewer for this important point. All participants had not received antibiotics, probiotics, or anti-inflammatory medications in the preceding 6 months, and none had known autoimmune or metabolic diseases. This information has been added to the Methods section (Line 449-452, page 22).

Regarding diet, lifestyle, non-antibiotic medications, and supplements: we did not systematically record these factors in this initial exploratory study. However, we did collect each participant's province and city of residence. The majority of participants in both groups were from Zhejiang Province (VLS: 37/43, 86.0%; control: 44/46, 95.7%).

This suggests that geographic and environmental factors that might influence the gut microbiota are broadly comparable between groups.

Nevertheless, we acknowledge that unmeasured factors such as daily diet, lifestyle habits, and use of non-antibiotic or over-the-counter medications could contribute to individual variability. Because these factors were not recorded, we were unable to adjust for them in our statistical analyses. We have now clearly stated this as a major limitation in the Discussion section (Line 301-307, page 14)

We hope this addresses the reviewer's concern.

4. The PCoA plot for 16S rRNA gene sequencing data may be modified to indicate what percentage of the variation is explained by each axis.

Response: We have added the percentage of variance explained by PC1 and PC2 in the axis labels in Figure 1D. The first two principal coordinates explained 12.16% and 10.16% of the total variation, respectively, indicating similar overall microbial community structure between groups.

5. In the metabolome profiling experiment, the PLS-DA analysis plot shows that very less variation in the data is explained by the PC1 (~7) and PC2 (~6) axes.

Response: We thank the reviewer for this important technical observation. We agree that the variance explained by the first two components (PC1/PC2) in the PLS-DA score plot appears relatively low. However, we would like to clarify that in PLS-DA, the primary goal is not to maximize the explained variance of the predictor matrix (X) but to find latent components that maximize the covariance between X and the class labels (Y). Therefore, the variance explained per component can be low, yet the model can still achieve excellent class separation and predictive performance.

To demonstrate that our PLS-DA models are valid despite the low variance explained, we have now calculated the cross-validated performance metrics. The models achieved a Q^2 (predictive ability) of 0.891 (positive mode) and 0.873 (negative mode), indicating high robustness and reproducibility of group separation, rather than random variation. We have added the Q^2 metric and this interpretation to the revised manuscript (Methods

and Results sections, Line 545-548, page 26, Line 179-181, page 9).

Moreover, low variance on the first two components is not uncommon in untargeted metabolomics of human serum, where the number of features (hundreds) far exceeds the number of samples (~40 per group).

6. How do the authors explain the separation along these axes? Have VIP scores been calculated to identify metabolites driving the separation between the groups?

Response: We greatly appreciate this constructive suggestion. The separation between VLS and control groups in the PLS-DA model reflects multivariate differences in metabolomic profiles between VLS and control groups, captured by latent components maximizing covariance between metabolite features and class labels. To identify metabolites contributing to group discrimination, we calculated Variable Importance in Projection (VIP) scores from the PLS-DA model. Metabolites with variable importance in projection (VIP) scores > 1 were considered to have a higher contribution to class separation and were further evaluated in combination with $P < 0.05$ and fold change > 1.5.

The observed separation was mainly attributed to subtle but consistent differences in serum metabolites involved in up-regulated metabolites in VLS, including myo-Inositol, azelaic acid, phenylpyruvic acid, and L-Serine, as well as down-regulated metabolites such as deoxycorticosterone acetate and guanidoacetic acid. These metabolites collectively contribute to the distinct metabolic signature of VLS. For clarity, we have now highlighted these metabolites in the volcano plot (Figure 4C) and listed all VIP-derived differential metabolites in Miscellaneous File 7.

We have added clear statements regarding VIP score calculation and its use in identifying key metabolites in the revised Methods and Results sections to ensure full transparency and methodological rigor. We hope this addresses the reviewer's concern (Line 182-184, page 9; Line 547-550, page 26).

7. The figure legends merely describe the figures, but do not convey the main findings/interpretations from the figures. Inclusion of detailed legends will make

figures self-explanatory.

Response: Thanks for the comment. All figure legends have been comprehensively revised according to the comment.

8. Access to the data generated in the study is restricted by a login step.

Response: We thank the reviewer for pointing out this concern. We have carefully verified the accessibility of our data in the Genome Sequence Archive (GSA). We would like to clarify that no login or registration is required to access the data. Readers can simply visit the GSA website at https://ngdc.cncb.ac.cn/gsa, enter the accession number CRA034514 in the search box, and then view and download all raw sequencing data freely.

To confirm this, we have tested the access procedure ourselves: (1) open the GSA homepage; (2) type "CRA034514" into the search field; (3) the dataset page appears with all files available for direct download without any login step. As stated on the GSA website, the data are "publicly accessible at https://ngdc.cncb.ac.cn/gsa"

9. In summary, there appear to be minor differences in the microbiome and metabolite profiles between the VLS and control groups in the age group of affected individuals. It may be useful to modify the manuscript accordingly.

Response: We thank the reviewer for this insightful comment. We agree that the differences in microbiome and metabolite profiles between VLS and control groups are modest. We have revised the manuscript accordingly to ensure a more conservative interpretation throughout the Results and Discussion sections. See point 2 response section.

Reviewer #2 (Comments for the Author):

In this manuscript, the authors performed 16S sequencing (feces) and untargeted metabolomics (serum) on a cohort of approx. 90 prepubertal girls with vulvar lichen sclerosus. The study is well designed and cohort size is sufficient. The authors analyzed

both microbiome and metabolomics data separately, as well as integreated both data types. The statistical analysis seems appropriate, the manuscript is well written and the results are interesting, and I believe both the data and the analysis will be of interest to the field. There are some comments that I think should be addressed to improve the manuscript:

General comments:

I think adding a table summarizing the two groups (healthy controls and prepubertal VLS) and detailing basic demographics would be helpful. It would be good to include the number of samples with 16S and metabolomics, age range, and any other relevant information, including statistics on comparison of the different demographics (such as age) between the groups.

Response: We thank the reviewer for the constructive suggestion. We have now added Miscellaneous File 2 summarizing the demographic characteristics of the two groups (healthy controls and prepubertal VLS patients), including sample sizes for 16S and metabolomics, age and weight distributions (median and Interquartile Range (IQR)), and p-values from Wilcoxon rank-sum tests comparing the two groups. As shown in Miscellaneous File 2, both age and weight differed significantly between groups ($p < 0.05$).

To rule out potential confounding effects, we re-analyzed both 16S and metabolomics data by including age and weight as covariates in the statistical models.

We have updated the Methods section accordingly (Line 493-497, page 24) and revised Miscellaneous File 2 to include the demographic comparison and a footnote describing covariate adjustment.

We believe these changes strengthen the validity of our conclusions. Thank you again for this important comment.

16S rRNA data processing: were samples rarified prior to alpha diversity analysis? if so, to what depth (please specify in the methods)? If not, rarefaction needs to be applied prior to analysis.

Response: We apologize for the omission. Yes, samples were rarified to a uniform depth of 15,000 reads per sample prior to alpha diversity analysis. This depth was chosen based on the rarefaction curves (Fig. S1A), which approached saturation for all samples. We have now clearly stated this in the Methods section (Line 486-488, page 23): "For alpha diversity analysis, samples were rarefied to a uniform depth of 15,000 reads per sample, which was the minimum sequencing depth across all samples after quality filtering."

Results:

L110: is it 33 healthy controls as stated here? or 45 healthy controls as stated in the abstract (line 48)? also 44 prepubertal VLS (as in the abstract) or 42? I see it is explained in the methods (that some samples had only 16S or only metabolomics), but would be good to clarify this in the results as well.

Response: We apologize for this confusion. The numbers are different because not all participants provided both feces and serum samples:

Total recruited: 43 VLS, 46 controls

16S rRNA sequencing: 42 VLS, 33 controls

Metabolomics: 39 VLS, 42 controls

We have clearly stated these sample sizes in the Results section and Miscellaneous File 1 to avoid ambiguity.

The revised text (Line 119-123, page 6): "A total of 89 participants (43 VLS patients and 46 healthy controls) were initially enrolled. After quality control, fecal samples from 75 participants (42 VLS, 33 controls) were successfully sequenced for the 16S rRNA gene, and serum samples from 81 participants (39 VLS, 42 controls) were profiled by LC-MS. Paired fecal and serum samples were available for 67 participants (38 VLS, 29 controls) for integrative analysis (Miscellaneous File 1)."

L124: " decreased Proteobacteria in VLS (P = 0.041, wilcoxon test)" - can you also provide the FDR corrected q-value?

Response: We have provided the corrected q-values in the revised manuscript and Table 1 (Line 143-147, page 7).

Table1 / Table2 - "P values were calculated via the Wilcoxon rank-sum test, with significance set at P < 0.05. Benjamini-Hochberg (BH) correction was used for multiple-testing adjustment." Are the values listed in the table the raw p-values or the FDR corrected q-values? Please clarify in the table legend (and if they are the raw p-values, please also provide the FDR corrected q-values in the table as well).

Response: We thank the reviewer for raising this important statistical point. In the revised manuscript, we have clarified in the table legends that the columns labelled "*P* value" contain raw P-values from the Wilcoxon rank-sum test, and the newly added "Adjusted q value" columns present the Benjamini-Hochberg false discovery rate (FDR) corrected q-values. The table legends now read: "P values were calculated via the Wilcoxon rank-sum test, with significance set at P < 0.05. Benjamini-Hochberg (BH) correction was used for multiple-testing adjustment; the adjusted q-values are provided in the adjacent column."

After FDR correction, the only taxon that remained statistically significant at the threshold of q < 0.05 was *Phocaeicola_A_858004* at the genus level (raw P=0.0041, corrected q = 0.0410). All other taxonomic differences, including those at the phylum level (e.g., *Firmicutes_A*: raw P=0.0213, q=0.2127) and the remaining genera (e.g., *Bacteroides_H*, *Dialister Fenollaria*, *Streptococcus*), did not reach significance after multiple-testing correction (q>0.05).

We appreciate the reviewer's rigorous statistical scrutiny. The lack of significance for most taxa after FDR correction is likely due to the relatively small effect sizes combined with the large number of taxa tested. Nevertheless, we would like to note that: These taxa were also identified by LEfSe (LDA score > 2.0, P < 0.05), which uses a different algorithm that is less sensitive to multiple testing when features have consistent directional changes. The direction of changes remains biologically coherent

aligns with functional pathway predictions (e.g., altered amino acid and lipid metabolism).

L200: "Guanidinoacetic acid was identified as a top feature" - It seems there are a lot of metabolites that show a stronger difference between the groups comapred to guanidinoacetic acid (both in fold change and p-value). Do you have any speculations as to why this metabolite was the top feature?

Response: This is an excellent observation. While guanidinoacetic acid did not have the largest fold-change or the smallest P-value, it was ranked as the top feature by the random forest model based on the mean decrease Gini index. This metric reflects the metabolite's contribution to classification accuracy when considered together with all other features in the model, rather than its univariate effect size. A metabolite with moderate but consistent discriminative power across many decision trees can outperform a metabolite with a large fold-change but higher variability. We have added a brief explanation in the Results (Line 229-231, page 11): "Although other metabolites showed larger fold-changes, guanidinoacetic acid was identified as the top feature by the random forest model based on its high mean decrease Gini index, indicating its strong contribution to classification accuracy when combined with other metabolites."

Methods:

L379: "Sample recruitment" - how were the controls recruited? Were they healthy volunteers? Or children with other conditions?

Response: The healthy control children were recruited from patients undergoing minor procedures at the same hospital, specifically those presenting for external genital nevus removal or redundant hymen surgery. They had no acute or chronic diseases, dermatological conditions, or other inflammatory disorders. This information has been added to the Methods section (Line 451-454, page 22): "Healthy controls were recruited from the same hospital from children undergoing external genital nevus removal or redundant hymen surgery. They had no history of anogenital diseases, autoimmune diseases, or any chronic inflammatory conditions."

L393: "written informed consent was obtained from each participant" - since the participants are children, was consent obtained from their parents or legal guardians? Please clarify.

Response: We apologize for the lack of clarity. We have revised the sentence (Line 464-465, page 22) to read: “All procedures were approved by the institutional ethics committee (2022-IRB-210), and written informed consent was obtained from each participant’s parent or legal guardian prior to sample collection.”

L424: Functional pathway prediction of microbiome - does not state FDR correction for multiple testing, whereas Fig3C the p-value (and in the legend) says "corrected". What correction is it?

Response: We apologize for the missing information. Benjamini–Hochberg correction was applied for multiple testing in the microbial functional pathway analysis. This has been added to the Methods section (Line 505-506, page 24) and Figure 3 legend.

L444: "Differential metabolites were defined based on P-value < 0.05" - How were the p-values calculated? Was there any correction for multiple testing? If so, please specify the method used and provide the corrected p-values (q-values) in the results as well.

Response: We have revised this section to provide complete information. The *P*-values were calculated using a two-tailed Student’s t-test. Then, we applied the Benjamini-Hochberg correction. We have added these details in Methods and Results (Line 182-184, page 9; Line 549-550, page 26), and the corrected p-values were provided in Miscellaneous File 5.

L463: "identified discriminant taxa with 100-fold cross-validation" - how was 100-fold cross-validation performed if only less than 100 samples were present?

Response: We thank the reviewer for pointing out this imprecise description. We

apologise for the confusion caused by the wording "100-fold cross-validation".

In fact, what we performed was not a 100-fold cross-validation (which would indeed require at least 100 samples to have one sample per fold). Instead, we used 100 iterations for cross validation. In each iteration, we randomly split the data into a training set (~70% of samples) and a test set (~30% of samples), trained a random forest model, and evaluated its performance. This process was repeated 100 times to obtain stable estimates of the AUC and the variable importance (Gini index).

We have revised the manuscript accordingly: the term "100-fold cross-validation" has been replaced with "100 iterations" in the revised text (Line 567, page 27). We thank the reviewer for helping us improve the accuracy of our description.

Please include in the methods section some more experimental details about the mass-spectrometry (type of mass-spectrometer used, sample preparation, number of ms2 spectra (was it on each sample? on pooled samples?) etc.). Also, were all metabolomics samples run in a single batch? If not, were samples randomized into batches? How were batch effects controlled for?

Response: We sincerely appreciate the reviewer's constructive suggestion. We have supplemented comprehensive experimental details for the metabolomic analysis, including sample preparation, LC-MS/MS conditions, data-dependent MS2 acquisition and quality control (QC) design in the Methods section (Line 507-530, page 24-25).

"Metabolite profiling

Serum samples were prepared as follows: after thawing on ice, each sample was vortexed for 1 min, and an appropriate volume (≤ 2 mL) was transferred to a 2 mL centrifuge tube. 400 μL of methanol was added, vortexed for 1 min, and centrifuged at 12,000 rpm for 10 min at 4 °C. The entire supernatant was collected, concentrated to dryness, and reconstituted in 150 μL of 80 % methanol/water containing 2-chlorophenylalanine (4 ppm, internal standard). The reconstituted solution was filtered through a 0.22 μm PTFE membrane prior to LC-MS analysis.

Chromatographic separation was performed on a Waters ACQUITY UPLC System equipped with an ACQUITY UPLC® HSS T3 column (2.1 × 100 mm, 1.8 μm) at 40 °C. The flow rate was 0.3 mL/min, and the injection volume was 2 μL. For positive ion mode, mobile phases consisted of (A1) 0.1 % formic acid in water and (B1) 0.1 % formic acid in acetonitrile, using a gradient program: 0–1 min, 8 % B1; 1–8 min, 8–98 % B1; 8–10 min, 98 % B1; 10–10.1 min, 98–8 % B1; 10.1–12 min, 8 % B1. For negative ion mode, mobile phases were (A2) 5 mM ammonium formate in water and (B2) acetonitrile with the same gradient profile.

Mass spectrometry was performed on a Thermo Q-Exactive Orbitrap mass spectrometer equipped with an electrospray ionization (ESI) source. Full MS scans were acquired over m/z 100–1000 at a resolution of 70,000. Data-dependent MS/MS spectra were collected for the top 3 ions per cycle at a resolution of 17,500 with normalized collision energy of 30 eV. Dynamic exclusion was used to reduce redundant MS/MS scans.

All study samples were analyzed in **a single batch** without interruption. A pooled quality control (QC) sample was prepared by mixing equal aliquots of all study samples and was injected **every 10 injections** to monitor system stability. Because the entire run was completed in one batch, no additional batch-effect correction was required; the frequent QC injections confirmed that retention time shifts and signal intensity drifts were negligible throughout the analysis.”

Data availability:

L289: "The raw sequence data reported in this paper have been deposited in the Genome Sequence Archive (Genomics, Proteomics & Bioinformatics 2025) in National Genomics Data Center (Nucleic Acids Res 2025)" - are "(Genomics, Proteomics & Bioinformatics 2025)" and "(Nucleic Acids Res 2025)" references? to what?

Could the authors also provide access to the metabolomics data (raw and/or processed) in a public repository? This would allow other researchers to validate the findings and perform further analyses.

Response: We apologize for the ambiguity. The phrases (Genomics, Proteomics &

Bioinformatics 2025) and (Nucleic Acids Res 2025) are citations of the journals where the database descriptions of the Genome Sequence Archive (GSA) and the National Genomics Data Center (NGDC) were published, not references to our own work. Specifically:

The GSA database description was published in Genomics, Proteomics & Bioinformatics in 2025.

The NGDC overall description was published in Nucleic Acids Research in 2025.

To avoid confusion, we have revised the sentence in the Data availability section to clearly indicate that these are database references. The revised text now reads:

"The raw 16Ssequence data reported in this paper have been deposited in the Genome Sequence Archive (GSA; for a description of the GSA database, see Genomics, Proteomics & Bioinformatics 2025) in National Genomics Data Center (NGDC; for a description of NGDC, see Nucleic Acids Res 2025), China National Center for Bioinformation / Beijing Institute of Genomics, Chinese Academy of Sciences (GSA: CRA034514) that are publicly accessible at https://ngdc.cncb.ac.cn/gsa."

In addition, we agree wholeheartedly that metabolomics data should be publicly available. In our original manuscript, we had already deposited the metabolomics data in OMIX, a public repository at the China National Center for Bioinformation (CNCB). The accession number is OMIX016393, and the data can be accessed directly at https://ngdc.cncb.ac.cn/omix (search for OMIX016393). We have now made this statement clearer in the revised Data availability section:

"The metabolomics data reported in this paper have been deposited in the OMIX, China National Center for Bioinformation / Beijing Institute of Genomics, Chinese Academy of Sciences (https://ngdc.cncb.ac.cn/omix: accession no.OMIX016393)."

We believe these revisions address the reviewer's concerns fully. All data (16S and metabolomics) are now publicly accessible without login barriers, enabling other researchers to validate and extend our findings.

Minor

L47: "its"->"the"

Response: We thank the reviewer. We have corrected “its” to “the” in the manuscript.

Supplemental figure S1B: Is it after rarefaction? Also, why are there negative values in the violin plot? Also, I do not see any samples in FigS1A with < 150ASVs, but in FigS1B it seems there are samples with < 150ASVs (mainly in the control group)? Also, what is the difference between FigS1B and Figure 1E? Do they show the same thing? If so, why are they different? If not, please clarify.

Response: We thank the reviewer for carefully examining the supplementary figure. We have made the following clarifications and corrections:

Fig. S1B is after rarefaction. We have added a note in the legend: “Boxplot comparing observed ASVs between control and VLS groups after rarefaction to 15,000 sequences per sample.” As for FigS1B it seems there are samples with < 150ASVs, we are sorry for the mistake. We have now corrected the figureS1B which is corresponding to the ASVs after rarefaction to 15,000 sequencing depth. Figure 1E shows the number of observed ASVs at the raw sequencing depth (not rarefied).

They are not identical because rarefaction normalizes sequencing depth; however, the relative differences between groups are preserved. We have now clarified this distinction in the legends of both figures.

To avoid any confusion, we have revised the legend of Fig. S1B to explicitly state: “Boxplot comparing observed ASVs between control and VLS groups after rarefaction to 15,000 sequences per sample.” Revised the legend of Fig. 1E to state: “Boxplot of observed ASVs before rarefaction (raw ASV counts).”

We appreciate the reviewer’s keen observation, which has helped us improve the accuracy and clarity of our figures. The revised figures and legends have been uploaded.

Re: Spectrum04058-25R1 (**Integrated Microbiome and Metabolome profiling in Prepubertal Girls with Vulvar Lichen Sclerosus**)

Dear Dr. Lan YU:

Your manuscript has been accepted, and I am forwarding it to the ASM production staff for publication. Your paper will first be checked to make sure all elements meet the technical requirements. ASM staff will contact you if anything needs to be revised before copyediting and production can begin. Otherwise, you will be notified when your proofs are ready to be viewed.

Data Availability: ASM policy requires that data be available to the public upon online posting of the article, so please verify all links to sequence records, if present, and make sure that each number retrieves the full record of the data. If a new accession number is not linked or a link is broken, provide production staff with the correct URL for the record. If the accession numbers for new data are not publicly accessible before the expected online posting of the article, publication may be delayed; please contact ASM production staff immediately with the expected release date.

Publication Fees: For information on publication fees and which article types have charges, please visit our website. We have partnered with Copyright Clearance Center (CCC) to collect author charges. If fees apply to your paper, you will receive a message from no-reply@copyright.com with further instructions. For questions related to paying charges through RightsLink, please contact CCC at ASM_Support@copyright.com or toll free at +1-877-622-5543. CCC makes every attempt to respond to all emails within 24 hours.

ASM Membership: Corresponding authors may join or renew ASM membership to obtain discounts on publication fees. Need to upgrade your membership level? Please contact Customer Service at Service@asmusa.org.

Embargo Policy: A press release may be issued as soon as the manuscript is posted on the Spectrum Latest Articles webpage. The corresponding author will receive an email with the subject line "ASM Journals Author Services Notification" when the article is available online.

The ASM Journals program strives for constant improvement in our submission and publication process. Please tell us how we can improve your experience by taking this quick Author Survey.

Thank you for submitting your paper to Spectrum.

Sincerely,
Xinzhao Tong
Editor
Microbiology Spectrum

Reviewer #2 (Comments for the Author):

The authors have addressed all comments. I believe this manuscript will help improve human knowledge and data.
