# Supplementary material for: Integrated microbiome and metabolome profiling in prepubertal girls with vulvar lichen sclerosus
Source: Microbiol Spectr. 2026 Aug 27;14(10):e04058-25. doi: 10.1128/spectrum.04058-25 (PMC13689091; doi:10.1128/spectrum.04058-25)
Supplement: Supplementary file 2 [file spectrum.04058-25-s0002.docx]

**Miscellaneous Files 1 Sample Metadata for 16S rRNA Sequencing and Metabolomics Analysis**

| Sample id | Group | Recruitment age | Disease duration | Weight | Analytical applications |
| --- | --- | --- | --- | --- | --- |
| VLS-1 | VLS | 5Y5M | 1Y | 17.5 | 16S、metabolomics |
| VLS-2 | VLS | 6Y4M | 6M | 23 | 16S、metabolomics |
| VLS-3 | VLS | 5Y11M | 6M | 20.5 | 16S、metabolomics |
| VLS-4 | VLS | 9Y3M | 1Y | 41.5 | 16S、metabolomics |
| VLS-5 | VLS | 7Y3M | 1M | 34 | 16S、metabolomics |
| VLS-6 | VLS | 5Y4M | 6M | 19 | 16S、metabolomics |
| VLS-7 | VLS | 4Y11M | 1Y | 20.5 | 16S、metabolomics |
| VLS-8 | VLS | 9Y | 1M | 36 | 16S、metabolomics |
| VLS-9 | VLS | 6Y1M | 1M | 18.5 | 16S、metabolomics |
| VLS-10 | VLS | 8Y8M | 4Y4M | 27.5 | 16S、metabolomics |
| VLS-11 | VLS | 5Y1M | 1Y | 18.5 | 16S、metabolomics |
| VLS-12 | VLS | 4Y11M | 3M | 18 | 16S、metabolomics |
| VLS-13 | VLS | 4Y8M | 2Y | 21 | 16S、metabolomics |
| VLS-14 | VLS | 5Y8M | 6M | 18.5 | 16S、metabolomics |
| VLS-16 | VLS | 5Y9M | 3M | 18.5 | 16S、metabolomics |
| VLS-17 | VLS | 5Y4M | 6M | 17.5 | 16S、metabolomics |
| VLS-18 | VLS | 8Y3M | 3Y | 40 | 16S、metabolomics |
| VLS-19 | VLS | 4Y5M | 1Y | 20.5 | 16S、metabolomics |
| VLS-20 | VLS | 8Y9M | 6M | 26.5 | 16S、metabolomics |
| VLS-21 | VLS | 9Y3M | 2Y | 28.5 | 16S、metabolomics |
| VLS-22 | VLS | 3Y5M | 6M | 17 | 16S |
| VLS-23 | VLS | 11Y3M | 15D | 41.5 | 16S、metabolomics |
| VLS-24 | VLS | 10Y | 1M | 35 | 16S、metabolomics |
| VLS-25 | VLS | 4Y3M | 1M | 19 | 16S、metabolomics |
| VLS-26 | VLS | 11Y9M | 3M | 50 | 16S |
| VLS-27 | VLS | 5Y | 15D | 18.5 | 16S、metabolomics |
| VLS-28 | VLS | 9Y4M | 6M | 41.5 | 16S |
| VLS-29 | VLS | 3Y3M | 15D | 14 | 16S、metabolomics |
| VLS-30 | VLS | 7Y1M | 3Y | 26.5 | 16S、metabolomics |
| VLS-31 | VLS | 7Y5M | 2M | 19.5 | 16S、metabolomics |
| VLS-32 | VLS | 4Y6M | 6M | 14.5 | 16S、metabolomics |
| VLS-33 | VLS | 7Y | 2Y | 20 | 16S、metabolomics |
| VLS-34 | VLS | 9Y4M | 4Y | 30.5 | 16S、metabolomics |
| VLS-35 | VLS | 8Y6M | 2Y | 26.5 | 16S、metabolomics |
| VLS-36 | VLS | 4Y7M | 1Y | 15 | 16S、metabolomics |
| VLS-37 | VLS | 6Y7M | 3Y | 20 | 16S、metabolomics |
| VLS-38 | VLS | 13Y3M | 6Y | 58.5 | 16S、metabolomics |
| VLS-39 | VLS | 4Y9M | 1M | 16.5 | 16S |
| VLS-40 | VLS | 6Y1M | 2D | 21 | 16S、metabolomics |
| VLS-41 | VLS | 8Y4M | 2D | 21.5 | 16S、metabolomics |
| VLS-42 | VLS | 4Y3M | 1W | 18 | 16S、metabolomics |
| VLS-43 | VLS | 9Y8M | 6M | 26.5 | 16S、metabolomics |
| VLS-44 | VLS | 5Y10M | 1Y | 19.5 | metabolomics |
| Control-1 | CONTROL | 8Y | - | 22 | 16S、metabolomics |
| Control-2 | CONTROL | 8Y7M | - | 30.5 | 16S、metabolomics |
| Control-3 | CONTROL | 6Y5M | - | 20.5 | 16S、metabolomics |
| Control-5 | CONTROL | 9Y | - | 23 | 16S、metabolomics |
| Control-9 | CONTROL | 12Y5M | - | 44 | 16S、metabolomics |
| Control-10 | CONTROL | 6Y7M | - | 24 | 16S、metabolomics |
| Control-11 | CONTROL | 10Y3M | - | 26 | 16S、metabolomics |
| Control-12 | CONTROL | 12Y10M | - | 36 | 16S、metabolomics |
| Control-16 | CONTROL | 11Y6M | - | 39 | 16S、metabolomics |
| Control-17 | CONTROL | 12Y9M | - | 50.5 | 16S |
| Control-18 | CONTROL | 10Y10M | - | 27 | 16S、metabolomics |
| Control-19 | CONTROL | 12Y7M | - | 38 | 16S、metabolomics |
| Control-20 | CONTROL | 7Y5M | - | 24 | 16S |
| Control-21 | CONTROL | 10Y6M | - | 32 | 16S、metabolomics |
| Control-22 | CONTROL | 9Y2M | - | 30 | 16S、metabolomics |
| Control-23 | CONTROL | 12Y6M | - | 46 | 16S、metabolomics |
| Control-24 | CONTROL | 10Y | - | 43 | 16S、metabolomics |
| Control-25 | CONTROL | 12Y10M | - | 49 | 16S、metabolomics |
| Control-26 | CONTROL | 11Y5M | - | 38 | 16S |
| Control-28 | CONTROL | 11Y8M | - | 36 | 16S、metabolomics |
| Control-29 | CONTROL | 10Y10M | - | 30 | 16S、metabolomics |
| Control-30 | CONTROL | 9Y6M | - | 31.3 | 16S、metabolomics |
| Control-31 | CONTROL | 11Y5M | - | 33 | 16S、metabolomics |
| Control-32 | CONTROL | 13Y2M | - | 47.5 | 16S、metabolomics |
| Control-33 | CONTROL | 12Y5M | - | 43 | 16S |
| Control-34 | CONTROL | 11Y7M | - | 50.5 | 16S、metabolomics |
| Control-36 | CONTROL | 3Y5M | - | 15 | 16S、metabolomics |
| Control-37 | CONTROL | 7Y3M | - | 19 | 16S、metabolomics |
| Control-38 | CONTROL | 4Y8M | - | 15.5 | 16S、metabolomics |
| Control-39 | CONTROL | 15Y4M | - | 55.5 | 16S、metabolomics |
| Control-43 | CONTROL | 8Y10M | - | 28.5 | 16S、metabolomics |
| Control-44 | CONTROL | 8Y11M | - | 24.5 | 16S、metabolomics |
| Control-45 | CONTROL | 5Y10M | - | 28 | 16S、metabolomics |
| Control-46 | CONTROL | 3Y5M | - | 15.5 | metabolomics |
| Control-47 | CONTROL | 3Y1M | - | 16 | metabolomics |
| Control-48 | CONTROL | 3Y10M | - | 16.5 | metabolomics |
| Control-49 | CONTROL | 8Y11M | - | 28 | metabolomics |
| Control-50 | CONTROL | 4Y4M | - | 17.5 | metabolomics |
| Control-51 | CONTROL | 6Y2M | - | 21 | metabolomics |
| Control-52 | CONTROL | 11Y5M | - | 38 | metabolomics |
| Control-53 | CONTROL | 9Y5M | - | 28.5 | metabolomics |
| Control-54 | CONTROL | 12Y5M | - | 43 | metabolomics |
| Control-55 | CONTROL | 9Y1M | - | 23.5 | metabolomics |
| Control-56 | CONTROL | 13Y5M | - | 47.5 | metabolomics |
| Control-57 | CONTROL | 13Y2M | - | 48.5 | metabolomics |
| Control-58 | CONTROL | 11Y7M | - | 35.5 | metabolomics |

| Characteristic | VLS Group (n=44) | Control Group (n=45) | P-value |
| --- | --- | --- | --- |
| Sex, female (%) | 44 (100%) | 45 (100%) | - |
| Age (years), median (IQR) | 6.5 (5.0–8.8) | 10.3 (7.4–12.0) | <0.001* |
| Age range (years) | 3.3–13.3 | 3.1–15.4 | - |
| Body weight (kg), median (IQR) | 20.0 (17.5–27.5) | 31.0 (23.0–43.0) | <0.001* |
| Disease duration (months), median (IQR) | 6.0 (1.0–12.0) | - | - |
| Disease duration range | 2 days – 6 years | - | - |
| Number of fecal samples (16S) | 42 | 33 | - |
| Number of serum samples (metabolomics) | 39 | 42 | - |
| Number of paired samples | 37 | 30 | - |
