# Supplementary material for: Integrated microbiome and metabolome profiling in prepubertal girls with vulvar lichen sclerosus
Source: Microbiol Spectr. 2026 Aug 27;14(10):e04058-25. doi: 10.1128/spectrum.04058-25 (PMC13689091; doi:10.1128/spectrum.04058-25)
Supplement: Supplementary file 3 [file spectrum.04058-25-s0003.docx]

**Miscellaneous File 2 Demographic and clinical characteristics of the study population**

| Characteristic | VLS (n=43) | CONTROL (n=46) | *P*-value* |
| --- | --- | --- | --- |
| Age (years), median (IQR) | 6.1 (4.8-8.9) | 10.1 (7.5-12.1) | <0.001 |
| Age range (years) | 3.3 -13.3 | 3.1 -15.4 | - |
| Body weight (kg), median (IQR) | 20.0 (17.5-28.5) | 31.3 (23.0-44.0) | <0.001 |
| Disease duration (months), median (IQR) | 6.0 (1.0-12.0) | - | - |
| Disease duration range† | 2 days -6 years | - | - |
| Number of fecal samples (16S) | 42 | 33 | - |
| Number of serum samples (metabolomics) | 39 | 42 | - |
| Number of paired samples (16S + metabolomics) | 38 | 29 | - |

*Wilcoxon rank‑sum test (two‑sided).
†Disease duration data are from the original clinical records (not present in the txt file, retained from previous table).
