# Supplementary material for: Integrated microbiome and metabolome profiling in prepubertal girls with vulvar lichen sclerosus
Source: Microbiol Spectr. 2026 Aug 27;14(10):e04058-25. doi: 10.1128/spectrum.04058-25 (PMC13689091; doi:10.1128/spectrum.04058-25)
Supplement: Supplementary file 4 [file spectrum.04058-25-s0004.docx]

**Miscellaneous File 3 Relative abundance of Microbial Communities at Phylum**

| **Phylum** | **Mean relative abundance** | | ***P* value** | **Adjusted *q* value** |
| --- | --- | --- | --- | --- |
|  | **VLS** | **Control** |  |  |
| *Actinobacteriota* | 0.029043 | 0.036101 | 0.478954 | 0.478954 |
| *Bacteroidota* | 0.488413 | 0.495665 | 0.133881 | 0.329771 |
| *Campylobacterota* | 0.005874 | 0.005039 | 0.167053 | 0.329771 |
| *Firmicutes_A* | 0.237369 | 0.199056 | **0.021267*** | 0.212674 |
| *Firmicutes_C* | 0.078392 | 0.075161 | 0.197862 | 0.329771 |
| *Firmicutes_D* | 0.038988 | 0.025566 | 0.104794 | 0.329771 |
| *Fusobacteriota* | 0.024038 | 0.019575 | 0.400405 | 0.444895 |
| *Proteobacteria* | 0.078642 | 0.12782 | 0.094637 | 0.329771 |
| *Verrucomicrobiota* | 0.004208 | 0.000719 | 0.303549 | 0.433642 |
| *Others* | 0.015032 | 0.015297 | 0.367094 | 0.444895 |

**P* values were calculated via the Wilcoxon rank-sum test, with significance set at *P* < 0.05. Benjamini-Hochberg (BH) correction was used for multiple-testing adjustment.
