# Supplementary material for: Integrated microbiome and metabolome profiling in prepubertal girls with vulvar lichen sclerosus
Source: Microbiol Spectr. 2026 Aug 27;14(10):e04058-25. doi: 10.1128/spectrum.04058-25 (PMC13689091; doi:10.1128/spectrum.04058-25)
Supplement: Supplementary file 5 [file spectrum.04058-25-s0005.docx]

**Miscellaneous File 4 Functional alterations of metabolic pathways compared VLS with control subjects**

| **Pathway** | **VLS: mean rel. freq. (%)** | **VLS: std. dev. (%)** | **control: mean rel. freq. (%)** | **control: std. dev. (%)** | **p-values** | **p-values (corrected)** | **Difference between means** | **95.0% lower CI** | **95.0% upper CI** |
| --- | --- | --- | --- | --- | --- | --- | --- | --- | --- |
| arginine, ornithine and proline interconversion | 0.097179 | 0.030071 | 0.073455 | 0.029645 | 0.001227 | 0.001227 | 0.023724 | 0.009686 | 0.037761 |
| chorismate biosynthesis I | 0.733746 | 0.111935 | 0.669154 | 0.112825 | 0.017481 | 0.017481 | 0.064592 | 0.011676 | 0.117508 |
| coenzyme A biosynthesis I | 0.795457 | 0.17611 | 0.691626 | 0.168736 | 0.012658 | 0.012658 | 0.10383 | 0.02291 | 0.184751 |
| superpathway of aromatic amino acid biosynthesis | 0.753202 | 0.110035 | 0.691283 | 0.113013 | 0.021706 | 0.021706 | 0.061919 | 0.009332 | 0.114505 |
| superpathway of fatty acid biosynthesis initiation (E. coli) | 0.20212 | 0.156655 | 0.282711 | 0.176708 | 0.046378 | 0.046378 | -0.08059 | -0.15985 | -0.00133 |
| gallate degradation II | 9.24E-05 | 0.000286 | 0.000603 | 0.001391 | 0.04889 | 0.04889 | -0.00051 | -0.00102 | -2.63E-06 |
| gallate degradation I | 4.73E-05 | 0.000269 | 0.000581 | 0.001399 | 0.040828 | 0.040828 | -0.00053 | -0.00104 | -2.36E-05 |
| superpathway of glycol metabolism and degradation | 0.05901 | 0.070022 | 0.10112 | 0.09097 | 0.034444 | 0.034444 | -0.04211 | -0.08103 | -0.00319 |
| methylgallate degradation | 0.000115 | 0.000357 | 0.000753 | 0.001736 | 0.048793 | 0.048793 | -0.00064 | -0.00127 | -3.57E-06 |
| superpathway of L-arginine and L-ornithine degradation | 1.038848 | 0.149375 | 0.963277 | 0.162175 | 0.044895 | 0.044895 | 0.07557 | 0.001772 | 0.149368 |
| TCA cycle IV (2-oxoglutarate decarboxylase) | 0.157795 | 0.125471 | 0.228754 | 0.133788 | 0.023977 | 0.023977 | -0.07096 | -0.13227 | -0.00965 |
| pantothenate and coenzyme A biosynthesis I | 0.600485 | 0.128205 | 0.542915 | 0.109157 | 0.04199 | 0.04199 | 0.057569 | 0.002142 | 0.112996 |
| superpathway of phospholipid biosynthesis I (bacteria) | 0.764334 | 0.12765 | 0.697143 | 0.129951 | 0.030535 | 0.030535 | 0.067191 | 0.006499 | 0.127884 |
| L-lysine biosynthesis II | 0.121098 | 0.097431 | 0.081169 | 0.065996 | 0.04087 | 0.04087 | 0.039929 | 0.001703 | 0.078155 |
| aerobic respiration I (cytochrome c) | 0.061666 | 0.042316 | 0.040186 | 0.026602 | 0.009988 | 0.009988 | 0.02148 | 0.005304 | 0.037657 |
| creatinine degradation II | 0.000229 | 0.000352 | 4.99E-05 | 0.000104 | 0.003231 | 0.003231 | 0.000179 | 6.29E-05 | 0.000296 |
| superpathway of L-methionine biosynthesis (by sulfhydrylation) | 0.141948 | 0.098364 | 0.194586 | 0.115794 | 0.043878 | 0.043878 | -0.05264 | -0.10379 | -0.00149 |
| CDP-diacylglycerol biosynthesis I | 0.865955 | 0.162657 | 0.781018 | 0.181488 | 0.041905 | 0.041905 | 0.084937 | 0.003206 | 0.166668 |
| urate biosynthesis/inosine 5'-phosphate degradation | 0.922999 | 0.199083 | 0.819267 | 0.21532 | 0.0386 | 0.0386 | 0.103731 | 0.005604 | 0.201858 |
| superpathway of menaquinol-9 biosynthesis | 0.114171 | 0.111713 | 0.169763 | 0.111754 | 0.038567 | 0.038567 | -0.05559 | -0.10818 | -0.00301 |
| superpathway of menaquinol-6 biosynthesis I | 0.114171 | 0.111713 | 0.169763 | 0.111754 | 0.038567 | 0.038567 | -0.05559 | -0.10818 | -0.00301 |
| superpathway of demethylmenaquinol-6 biosynthesis I | 0.092878 | 0.099843 | 0.141097 | 0.103972 | 0.049471 | 0.049471 | -0.04822 | -0.09632 | -0.00011 |
| superpathway of demethylmenaquinol-9 biosynthesis | 0.092878 | 0.099843 | 0.141097 | 0.103972 | 0.049471 | 0.049471 | -0.04822 | -0.09632 | -0.00011 |
| superpathway of menaquinol-10 biosynthesis | 0.114171 | 0.111713 | 0.169763 | 0.111754 | 0.038567 | 0.038567 | -0.05559 | -0.10818 | -0.00301 |
| stearate biosynthesis II (bacteria and plants) | 0.23546 | 0.183998 | 0.335191 | 0.216005 | 0.040964 | 0.040964 | -0.09973 | -0.19524 | -0.00423 |
| palmitoleate biosynthesis I (from (5Z)-dodec-5-enoate) | 0.230691 | 0.178605 | 0.325809 | 0.207403 | 0.043056 | 0.043056 | -0.09512 | -0.18717 | -0.00306 |
| adenine and adenosine salvage III | 0.869492 | 0.204564 | 0.749069 | 0.215036 | 0.018 | 0.018 | 0.120423 | 0.021313 | 0.219534 |
| superpathway of L-tryptophan biosynthesis | 0.152363 | 0.146223 | 0.228569 | 0.145084 | 0.029769 | 0.029769 | -0.07621 | -0.14471 | -0.0077 |
| cob(II)yrinate a,c-diamide biosynthesis I (early cobalt insertion) | 0.191413 | 0.091116 | 0.14732 | 0.086877 | 0.038784 | 0.038784 | 0.044093 | 0.002337 | 0.085849 |
| aromatic biogenic amine degradation (bacteria) | 0.000558 | 0.001044 | 0.00017 | 0.00029 | 0.02751 | 0.02751 | 0.000388 | 4.49E-05 | 0.000732 |
| oleate biosynthesis IV (anaerobic) | 0.25178 | 0.186963 | 0.347721 | 0.214752 | 0.049415 | 0.049415 | -0.09594 | -0.19163 | -0.00025 |
| CDP-diacylglycerol biosynthesis II | 0.865955 | 0.162657 | 0.781018 | 0.181488 | 0.041905 | 0.041905 | 0.084937 | 0.003206 | 0.166668 |
| mycolate biosynthesis | 0.253375 | 0.189639 | 0.350847 | 0.216118 | 0.047961 | 0.047961 | -0.09747 | -0.19404 | -0.0009 |
