# Supplementary material for: Integrated microbiome and metabolome profiling in prepubertal girls with vulvar lichen sclerosus
Source: Microbiol Spectr. 2026 Aug 27;14(10):e04058-25. doi: 10.1128/spectrum.04058-25 (PMC13689091; doi:10.1128/spectrum.04058-25)
Supplement: Supplementary file 6 [file spectrum.04058-25-s0006.docx]

**Miscellaneous File 5 Metabolites identified in positive and negative mode**

| **ID** | **name** | **mz** | **rt** | **exact_mass** | **ppm** | **library** | **posneg** |
| --- | --- | --- | --- | --- | --- | --- | --- |
| M102T75_1 | 2-Iminobutanoate | 102.0552 | 75.2 | 101.0477 | 2.194895 | MetaDNA | pos |
| M151T245 | Phenylglyoxylic acid | 151.0345 | 245.1 | 150.0317 | 29.63473 | MetaDNA | pos |
| M161T47 | L-Carnitinamide | 161.1284 | 46.6 | 161.129 | 3.723724 | MetaDNA | pos |
| M348T329 | 17alpha,21-Dihydroxypregnenolone | 348.2336 | 328.8 | 348.2301 | 10.05083 | MetaDNA | pos |
| M364T326 | Dihydrocortisol | 364.2188 | 326.5 | 364.225 | 17.02244 | MetaDNA | pos |
| M381T607 | 24-Methylenecholesterol | 381.3546 | 606.9 | 398.3549 | 18.70735 | MetaDNA | pos |
| M118T44_2 | L-Aspartate-semialdehyde | 118.0474 | 44 | 117.0426 | 20.97419 | MetaDNA | pos |
| M179T273 | 2-Oxo-4-phenylbutyric acid | 179.0702 | 273.4 | 178.063 | 0.424414 | MetaDNA | pos |
| M185T42_1 | Phosphohydroxypyruvic acid | 184.9847 | 41.5 | 183.9773 | 0.670326 | MetaDNA | pos |
| M300T391 | 9-cis-Retinoic acid | 300.2166 | 391.5 | 300.2089 | 25.64881 | MetaDNA | pos |
| M660T476 | Coproporphyrinogen III | 660.3199 | 476.4 | 660.3159 | 6.057707 | MetaDNA | pos |
| M147T92 | 3-Aminopentanedioate | 147.0497 | 91.8 | 147.0532 | 23.80091 | MetaDNA | pos |
| M437T483 | 27-Deoxy-5b-cyprinol | 437.3722 | 482.6 | 436.3553 | 22.00463 | MetaDNA | pos |
| M385T352 | 7a-Hydroxy-5b-cholestan-3-one | 385.3435 | 352.4 | 402.3498 | 2.942838 | MetaDNA | pos |
| M350T243 | Prostaglandin E3 | 350.2052 | 243.4 | 350.2093 | 11.70728 | MetaDNA | pos |
| M377T689 | Ergosta-5,7,22,24(28)-tetraen-3beta-ol | 377.3204 | 688.8 | 394.3236 | 11.22136 | MetaDNA | pos |
| M355T618 | Cholestane | 355.3761 | 618.3 | 372.3756 | 22.32614 | MetaDNA | pos |
| M399T600 | Stigmastanol | 399.3936 | 600 | 416.4018 | 1.917904 | MetaDNA | pos |
| M378T304 | Cornudentanone | 378.2367 | 304 | 378.2406 | 10.3109 | MetaDNA | pos |
| M407T537 | Ardisianone | 407.2738 | 536.9 | 406.2719 | 13.19979 | MetaDNA | pos |
| M286T420 | Neoabietal | 286.2377 | 420.3 | 286.2297 | 27.94958 | MetaDNA | pos |
| M352T384 | Lipoxin A4 | 352.2238 | 384 | 352.225 | 3.406913 | MetaDNA | pos |
| M415T376 | 3beta,5beta-Ketotriol | 415.3195 | 375.8 | 432.324 | 7.064491 | MetaDNA | pos |
| M417T558 | 3beta,5beta-Ketodiol | 417.3315 | 557.6 | 416.329 | 11.44401 | MetaDNA | pos |
| M102T276 | Isovaleric acid | 102.0909 | 276.1 | 102.0681 | 6.543122 | BioDeepDB | pos |
| M102T86 | 2-Ketobutyric acid | 102.0341 | 86.2 | 102.0317 | 0.62876 | BioDeepDB | pos |
| M102T58 | Valeric acid | 102.0553 | 58.2 | 102.0681 | 0.469596 | BioDeepDB | pos |
| M104T51 | gamma-Aminobutyric acid | 104.1072 | 51.3 | 103.0633 | 1.764797 | BioDeepDB | pos |
| M122T58 | (S)-1-Phenylethanol | 122.0966 | 58.3 | 122.0732 | 1.471883 | BioDeepDB | pos |
| M109T54 | m-Cresol | 109.1015 | 54.2 | 108.0575 | 0.013426 | BioDeepDB | pos |
| M110T385 | Hydroquinone | 110.0203 | 384.9 | 110.0368 | 0.016532 | BioDeepDB | pos |
| M114T53 | Creatinine | 114.0664 | 52.5 | 113.0589 | 0.331616 | BioDeepDB | pos |
| M116T54_1 | L-Proline | 116.0708 | 53.5 | 115.0633 | 0.085973 | BioDeepDB | pos |
| M118T71 | L-Valine | 118.0863 | 71.4 | 117.079 | 0.203241 | BioDeepDB | pos |
| M119T438 | N,N-Dimethyl-1,4-phenylenediamine | 119.0852 | 437.9 | 136.1 | 2.02871 | BioDeepDB | pos |
| M123T80 | Benzoate | 123.0404 | 80.4 | 122.0368 | 29.87637 | BioDeepDB | pos |
| M126T198 | Thymine | 125.986 | 197.9 | 126.0429 | 5.692887 | BioDeepDB | pos |
| M130T74 | Pipecolic acid | 130.0875 | 74.3 | 129.079 | 6.189307 | BioDeepDB | pos |
| M130T332 | Heptanoic acid | 130.0652 | 331.9 | 130.0994 | 1.870869 | BioDeepDB | pos |
| M131T171 | Leucine | 130.9671 | 170.7 | 131.0946 | 2.664038 | BioDeepDB | pos |
| M131T96 | cis-4-Hydroxy-L-proline | 130.9666 | 95.9 | 131.0582 | 0.244389 | BioDeepDB | pos |
| M132T53 | Creatine | 132.0767 | 52.6 | 131.0695 | 0.168273 | BioDeepDB | pos |
| M132T75 | L-Isoleucine | 132.0544 | 75.2 | 131.0946 | 21.58941 | BioDeepDB | pos |
| M133T61 | L-Asparagine | 133.1053 | 61.5 | 132.0535 | 13.63501 | BioDeepDB | pos |
| M135T572 | Perillyl alcohol | 135.1167 | 572.3 | 152.1201 | 5.180707 | BioDeepDB | pos |
| M137T75_1 | 2-Aminobenzoic acid | 137.0457 | 75 | 137.0477 | 14.59367 | BioDeepDB | pos |
| M139T310 | 4-Hydroxybenzoic acid | 139.0173 | 309.6 | 138.0317 | 5.403266 | BioDeepDB | pos |
| M139T199 | Uracil 5-carboxylate | 139.0124 | 199 | 156.0171 | 19.66731 | BioDeepDB | pos |
| M142T41 | O-Phosphoethanolamine | 141.9586 | 40.8 | 141.0191 | 0.557904 | BioDeepDB | pos |
| M145T663 | Anabasine | 144.9818 | 662.6 | 162.1157 | 4.078839 | BioDeepDB | pos |
| M146T196 | L-Lysine | 146.0599 | 196.5 | 146.1055 | 1.607859 | BioDeepDB | pos |
| M147T52 | L-Glutamine | 147.0762 | 52.3 | 146.0691 | 1.196659 | BioDeepDB | pos |
| M149T521 | 3-Methyladenine | 149.0232 | 521.4 | 149.0701 | 0.761305 | BioDeepDB | pos |
| M151T155 | D-Ribose | 151.0345 | 154.7 | 150.0528 | 2.988558 | BioDeepDB | pos |
| M151T271 | D-Lyxose | 151.0345 | 270.8 | 150.0528 | 1.819321 | BioDeepDB | pos |
| M152T51 | Mandelic acid | 152.0219 | 51.2 | 152.0473 | 1.853145 | BioDeepDB | pos |
| M155T382 | 2,3-Butanediol | 154.9901 | 382.5 | 154.0122 | 0.506536 | BioDeepDB | pos |
| M156T51 | L-Histidine | 156.0418 | 50.8 | 155.0695 | 12.65405 | BioDeepDB | pos |
| M175T69 | Shikimic acid | 175.0313 | 68.8 | 174.0528 | 21.6729 | BioDeepDB | pos |
| M159T194 | 4,5-Dihydroorotic acid | 158.9603 | 193.5 | 158.0328 | 5.738277 | BioDeepDB | pos |
| M158T400 | Pelargonic acid | 158.154 | 399.7 | 158.1307 | 2.656647 | BioDeepDB | pos |
| M177T97 | Ascorbate | 176.9718 | 97.2 | 176.0321 | 1.734364 | BioDeepDB | pos |
| M160T93 | Pimelic acid | 160.0757 | 93.1 | 160.0736 | 13.11879 | BioDeepDB | pos |
| M163T363 | Acetylcysteine | 163.039 | 363 | 163.0303 | 0.476934 | BioDeepDB | pos |
| M166T116 | L-Phenylalanine | 166.0864 | 116 | 165.079 | 0.628828 | BioDeepDB | pos |
| M167T293 | Quinolinic acid | 167.0122 | 293.2 | 167.0219 | 4.244813 | BioDeepDB | pos |
| M168T131 | L-4-Hydroxyphenylglycine | 168.0634 | 131.1 | 167.0582 | 12.35248 | BioDeepDB | pos |
| M170T317 | Norepinephrine | 169.9772 | 317 | 169.0739 | 1.044141 | BioDeepDB | pos |
| M170T38 | Pyridoxine | 169.9976 | 38.5 | 169.0739 | 22.01663 | BioDeepDB | pos |
| M171T196 | 3,4-Dihydroxyphenylglycol | 171.0634 | 196 | 170.0579 | 10.38212 | BioDeepDB | pos |
| M170T460 | 1-Methylhistidine | 170.0962 | 459.8 | 169.0851 | 22.4814 | BioDeepDB | pos |
| M171T39 | Azelaic acid | 170.9799 | 38.7 | 188.1049 | 29.02845 | BioDeepDB | pos |
| M171T317 | 1-Hydroxy-2-naphthoate | 171.1493 | 317.1 | 188.0473 | 2.453969 | BioDeepDB | pos |
| M172T317_1 | 3-Dehydroshikimate | 171.9929 | 317.1 | 172.0372 | 0.750804 | BioDeepDB | pos |
| M175T47 | L-Arginine | 175.1189 | 46.6 | 174.1117 | 0.29165 | BioDeepDB | pos |
| M175T687 | N-Methyltryptamine | 175.1225 | 686.6 | 174.1157 | 2.718097 | BioDeepDB | pos |
| M177T235 | N-Formyl-L-methionine | 177.0498 | 234.7 | 177.046 | 21.46289 | BioDeepDB | pos |
| M177T121 | Methyl beta-D-galactoside | 176.9714 | 121.2 | 194.079 | 1.839076 | BioDeepDB | pos |
| M180T237 | Theophylline | 180.0654 | 236.7 | 180.0647 | 3.887476 | BioDeepDB | pos |
| M181T132 | D-Fructose | 181.072 | 132.3 | 180.0634 | 7.312008 | BioDeepDB | pos |
| M181T421 | myo-Inositol | 181.0147 | 421.2 | 180.0634 | 3.488095 | BioDeepDB | pos |
| M183T40 | 4-Pyridoxic acid | 182.984 | 40 | 183.0532 | 7.423305 | BioDeepDB | pos |
| M200T269 | Dodecanoic acid | 199.9863 | 269.2 | 200.1776 | 4.910266 | BioDeepDB | pos |
| M184T142 | 3,4-Dihydroxymandelic acid | 184.1696 | 142 | 184.037 | 0.847576 | BioDeepDB | pos |
| M187T204 | Undecanoic acid | 186.9561 | 204.2 | 186.162 | 1.162159 | BioDeepDB | pos |
| M188T197 | N-Alpha-acetyllysine | 188.0707 | 196.7 | 188.1161 | 0.178896 | BioDeepDB | pos |
| M190T521 | N-Acetylglutamic acid | 190.0496 | 521.5 | 189.0637 | 0.086159 | BioDeepDB | pos |
| M195T120 | D-Glucuronic Acid | 195.1227 | 120.5 | 194.043 | 0.054057 | BioDeepDB | pos |
| M197T358 | Gluconic acid | 197.1536 | 357.7 | 196.0583 | 2.663533 | BioDeepDB | pos |
| M198T604 | Vanillylmandelic acid | 198.1849 | 603.7 | 198.0528 | 0.241388 | BioDeepDB | pos |
| M205T197 | L-Tryptophan | 205.0974 | 196.6 | 204.0899 | 0.73362 | BioDeepDB | pos |
| M227T604_1 | Deoxycytidine | 227.1637 | 603.8 | 227.0906 | 4.618456 | BioDeepDB | pos |
| M228T603 | Deoxyuridine | 228.1956 | 603.2 | 228.0746 | 0.457193 | BioDeepDB | pos |
| M216T103 | 12-Hydroxydodecanoic acid | 216.1958 | 103.3 | 216.1725 | 1.891276 | BioDeepDB | pos |
| M224T278 | Hydroxykynurenine | 224.1256 | 277.7 | 224.0797 | 11.2973 | BioDeepDB | pos |
| M226T536 | Porphobilinogen | 226.18 | 536.2 | 226.0954 | 2.126435 | BioDeepDB | pos |
| M230T379 | D-Ribose 5-phosphate | 230.2477 | 379.5 | 230.0192 | 0.181106 | BioDeepDB | pos |
| M231T380 | Butyrylcarnitine | 231.2512 | 379.8 | 231.1471 | 0.977295 | BioDeepDB | pos |
| M240T110 | Anserine | 240.1021 | 109.7 | 240.1222 | 24.92643 | BioDeepDB | pos |
| M272T233 | 17a-Estradiol | 272.185 | 232.9 | 272.1776 | 0.417713 | BioDeepDB | pos |
| M278T236 | Gamma-Linolenic acid | 278.1852 | 236 | 278.2246 | 0.521155 | BioDeepDB | pos |
| M281T507 | Linoleic acid | 281.2472 | 507 | 280.2402 | 0.981343 | BioDeepDB | pos |
| M282T617 | (6Z)-Octadecenoic acid | 282.278 | 616.5 | 282.2559 | 0.857125 | BioDeepDB | pos |
| M267T242 | 2'-Deoxyguanosine | 267.0993 | 242 | 267.097 | 8.61103 | BioDeepDB | pos |
| M284T238 | Stearic acid | 284.1853 | 237.6 | 284.2715 | 1.260853 | BioDeepDB | pos |
| M288T392 | Dehydroepiandrosterone | 288.2892 | 391.5 | 288.2089 | 2.275344 | BioDeepDB | pos |
| M271T596 | Baicalein | 271.2816 | 596.1 | 270.0528 | 25.25141 | BioDeepDB | pos |
| M279T477 | Alpha-Linolenic acid | 279.2316 | 477.4 | 278.2246 | 0.988427 | BioDeepDB | pos |
| M282T597 | 1-Methyladenosine | 282.2212 | 596.8 | 281.1124 | 9.113668 | BioDeepDB | pos |
| M282T590 | Oleic acid | 282.278 | 589.7 | 282.2559 | 1.639683 | BioDeepDB | pos |
| M302T412 | Sphinganine | 302.3056 | 412.3 | 301.2981 | 0.602546 | BioDeepDB | pos |
| M273T666 | Androsterone | 273.2251 | 666.2 | 290.2246 | 14.26396 | BioDeepDB | pos |
| M295T48 | Indican | 294.9378 | 47.9 | 295.1056 | 3.535571 | BioDeepDB | pos |
| M301T174 | all-trans-Retinoic acid | 301.1382 | 174.5 | 300.2089 | 6.836692 | BioDeepDB | pos |
| M312T682 | Arachidic acid | 312.3244 | 681.8 | 312.3028 | 2.484596 | BioDeepDB | pos |
| M321T594 | Pregnanediol | 321.3138 | 594.5 | 320.2715 | 3.973935 | BioDeepDB | pos |
| M343T380 | Sucrose | 343.2963 | 379.8 | 342.1162 | 4.479767 | BioDeepDB | pos |
| M328T240 | Docosahexaenoic acid | 328.1986 | 240.3 | 328.2402 | 1.650951 | BioDeepDB | pos |
| M347T329 | Cortexolone | 347.2298 | 329.1 | 346.2144 | 4.109082 | BioDeepDB | pos |
| M338T573 | Erucic acid | 338.3389 | 572.8 | 338.3185 | 1.253181 | BioDeepDB | pos |
| M360T239 | Rosmarinic acid | 360.2369 | 238.5 | 360.0845 | 0.022094 | BioDeepDB | pos |
| M362T380 | Thyrotropin releasing hormone | 362.3258 | 379.8 | 362.1703 | 3.047801 | BioDeepDB | pos |
| M376T431 | Riboflavin | 376.2578 | 431.5 | 376.1383 | 3.485797 | BioDeepDB | pos |
| M376T545 | Lithocholic acid | 376.3186 | 545.4 | 376.2977 | 0.201957 | BioDeepDB | pos |
| M385T518 | Delta-Tocopherol | 385.2812 | 518.1 | 402.3498 | 29.10056 | BioDeepDB | pos |
| M385T562 | Vitamin D3 | 385.2917 | 562.4 | 384.3392 | 0.27133 | BioDeepDB | pos |
| M391T685 | S-Hexyl-glutathione | 391.2825 | 684.5 | 391.1777 | 2.584241 | BioDeepDB | pos |
| M393T229 | Ursodeoxycholic acid | 393.2092 | 228.9 | 392.2927 | 0.60525 | BioDeepDB | pos |
| M398T432 | S-Adenosylmethionine | 398.2396 | 431.6 | 398.137 | 3.066344 | BioDeepDB | pos |
| M413T618 | alpha-Tocopherol | 413.3235 | 617.7 | 430.3811 | 1.23375 | BioDeepDB | pos |
| M432T438 | Isovitexin | 432.2363 | 437.9 | 432.1056 | 6.271375 | BioDeepDB | pos |
| M585T515 | Bilirubin | 585.2678 | 514.8 | 584.2635 | 3.770466 | BioDeepDB | pos |
| M104T486 | Choline | 104.1066 | 485.7 | 104.1075 | 3.842216 | BioDeepDB | pos |
| M118T198 | Indole | 118.0657 | 198.2 | 117.0578 | 5.081916 | BioDeepDB | pos |
| M137T623 | Phenelzine | 137.1073 | 623.5 | 136.1 | 0.031793 | BioDeepDB | pos |
| M147T662 | Dimethylbenzimidazole | 147.0919 | 661.8 | 146.0844 | 1.359694 | BioDeepDB | pos |
| M148T689 | Pseudoephedrine | 148.1119 | 688.9 | 165.1154 | 26.561 | BioDeepDB | pos |
| M161T687 | Anatabine | 161.1074 | 686.6 | 160.1 | 0.620704 | BioDeepDB | pos |
| M184T486 | Phosphorylcholine | 184.0718 | 486.5 | 184.0739 | 8.148994 | BioDeepDB | pos |
| M231T615_1 | Dodecanedioic acid | 231.1589 | 615.1 | 230.1518 | 0.761381 | BioDeepDB | pos |
| M219T329 | Capsidiol | 219.1742 | 329.1 | 236.1776 | 3.193807 | BioDeepDB | pos |
| M282T547 | Oleamide | 282.2787 | 546.7 | 281.2719 | 1.417039 | BioDeepDB | pos |
| M273T250 | Sotalol | 273.1343 | 249.6 | 272.1195 | 27.54689 | BioDeepDB | pos |
| M279T491 | 13S-hydroxyoctadecadienoic acid | 279.2314 | 491.3 | 296.2351 | 3.581259 | BioDeepDB | pos |
| M279T527 | Ethyl oleate | 279.2677 | 526.7 | 296.2715 | 13.0126 | BioDeepDB | pos |
| M291T511 | Sclareol | 291.2676 | 511 | 308.2715 | 4.119923 | BioDeepDB | pos |
| M299T509 | Sphingosine | 299.2771 | 508.9 | 299.2824 | 17.70934 | BioDeepDB | pos |
| M383T657 | Campesterol | 383.3697 | 657.3 | 400.3705 | 4.956052 | BioDeepDB | pos |
| M385T556 | 7-Dehydrocholesterol | 385.3492 | 555.9 | 384.3392 | 7.006632 | BioDeepDB | pos |
| M386T522 | Risperidone | 386.2656 | 522 | 385.2478 | 27.18337 | BioDeepDB | pos |
| M395T599 | alpha-Spinasterol | 395.3626 | 599.2 | 412.3705 | 1.178665 | BioDeepDB | pos |
| M397T581 | beta-Sitosterol | 397.3779 | 580.6 | 414.3862 | 2.179286 | BioDeepDB | pos |
| M412T596 | Stigmasterol | 412.3769 | 596.2 | 412.3705 | 15.51978 | BioDeepDB | pos |
| M496T486_1 | 1-palmitoylglycerophosphocholine | 496.3367 | 485.7 | 496.3403 | 6.24576 | BioDeepDB | pos |
| M107T298 | O-Toluidine | 107.0706 | 297.7 | 107.0735 | 27.08493 | MoNA | pos |
| M279T521 | Dibutyl phthalate | 279.1586 | 521.3 | 278.1518 | 1.705124 | MoNA | pos |
| M363T326 | Cortisol | 363.2154 | 326.2 | 362.2093 | 3.237748 | MoNA | pos |
| M185T272 | Phosphoserine | 185.0229 | 271.7 | 185.0089 | 2.154837 | BioDeepDB | pos |
| M175T386 | Gramine | 175.1233 | 386.5 | 174.1157 | 1.850125 | MoNA | pos |
| M205T57 | O-Acetylcarnitine | 205.126 | 56.8 | 204.1236 | 23.77076 | BioDeepDB | pos |
| M284T656 | Octadecanamide | 284.2943 | 656 | 283.2875 | 1.674321 | GNPS | pos |
| M147T537 | Myosmine | 147.0911 | 537.2 | 146.0844 | 3.904519 | MoNA | pos |
| M161T662 | Tolazoline | 161.1075 | 661.7 | 160.1 | 1.090055 | MoNA | pos |
| M186T57 | Ecgonine | 186.1124 | 56.8 | 185.1052 | 0.372571 | MoNA | pos |
| M218T86 | Propionylcarnitine | 218.1388 | 86.5 | 217.1314 | 0.531368 | MoNA | pos |
| M247T217 | Lenticin | 247.1442 | 216.8 | 246.1368 | 0.389182 | MoNA | pos |
| M105T386 | L-Serine | 105.0426 | 386.4 | 105.0426 | 0 | BioDeepDB | pos |
| M122T593 | N,N-Dimethylaniline | 122.0962 | 593.2 | 121.0891 | 1.441486 | mzCloud | pos |
| M165T75 | 2-Hydroxycinnamic acid | 165.0549 | 75.2 | 164.0473 | 0.010614 | BioDeepDB | pos |
| M264T411 | Tramadol | 264.1931 | 411.3 | 263.1885 | 10.12895 | mzCloud | pos |
| M250T385 | Alprenolol | 250.178 | 384.8 | 249.1729 | 8.613822 | mzCloud | pos |
| M251T564 | OPEO | 251.2002 | 564 | 250.1933 | 1.417514 | mzCloud | pos |
| M259T75 | (5-L-Glutamyl)-L-glutamate | 259.0925 | 75.2 | 276.0958 | 15.95569 | mzCloud | pos |
| M259T250 | Propranolol | 259.1518 | 250.5 | 259.1572 | 20.83721 | mzCloud | pos |
| M265T233 | Phenylacetylglutamine | 265.1171 | 233.3 | 264.111 | 4.435776 | mzCloud | pos |
| M307T522 | Dihomo-gamma-linolenate | 307.2628 | 522.5 | 306.2559 | 1.223708 | mzCloud | pos |
| M308T461 | 10-Nitrolinoleic acid | 308.219 | 461 | 325.2253 | 3.679202 | mzCloud | pos |
| M326T543 | Oleoylethanolamide | 326.3049 | 542.6 | 325.2981 | 1.458758 | mzCloud | pos |
| M318T378 | Phytosphingosine | 318.2998 | 377.8 | 317.293 | 1.495445 | mzCloud | pos |
| M321T281 | Mycophenolate | 321.1302 | 281.5 | 320.126 | 9.578669 | mzCloud | pos |
| M360T259 | Etoxazole | 360.1745 | 259.3 | 359.1697 | 6.874446 | mzCloud | pos |
| M399T510 | Tris(butoxyethyl)phosphate | 399.2498 | 509.8 | 398.2433 | 1.943645 | mzCloud | pos |
| M457T493 | Ursolic acid | 457.3618 | 492.8 | 456.3603 | 12.62895 | mzCloud | pos |
| M100T121 | 2-Pyrrolidinone | 100.0761 | 121.3 | 99.0684 | 0.107686 | BioDeepDB | pos |
| M105T668 | D-Serine | 105.0424 | 668.3 | 105.0426 | 0.002209 | BioDeepDB | pos |
| M107T116 | Benzaldehyde | 107.0494 | 116 | 106.0419 | 0.000391 | BioDeepDB | pos |
| M118T349 | L-2,4-diaminobutyric acid | 118.0659 | 349 | 118.0742 | 1.655849 | BioDeepDB | pos |
| M119T75 | 2-Methylserine | 119.0492 | 75.2 | 119.0582 | 0.642856 | BioDeepDB | pos |
| M121T54 | (S)-2-Phenyloxirane | 121.065 | 54.2 | 120.0575 | 1.850246 | BioDeepDB | pos |
| M130T306 | 6-Hydroxyhexan-6-olide | 130.0652 | 305.7 | 130.063 | 0.001147 | BioDeepDB | pos |
| M132T58 | 5-Aminolevulinic acid | 132.0647 | 57.7 | 131.0582 | 5.875908 | BioDeepDB | pos |
| M132T270 | 6-Hydroxyhexanoic acid | 132.0807 | 270.2 | 132.0786 | 2.254606 | BioDeepDB | pos |
| M132T197 | (S)-4-Amino-5-oxopentanoate | 132.0807 | 196.7 | 131.0582 | 26.84726 | BioDeepDB | pos |
| M137T57 | allopurinol | 137.0457 | 57.1 | 136.0385 | 0.045821 | BioDeepDB | pos |
| M138T64 | p-Aminobenzoic acid | 138.0534 | 63.9 | 137.0477 | 9.768214 | BioDeepDB | pos |
| M140T32 | Acetylphosphate | 139.9873 | 32.3 | 139.9875 | 1.428701 | BioDeepDB | pos |
| M146T60 | 4-Oxoglutaramate | 146.043 | 59.6 | 145.0375 | 12.1608 | BioDeepDB | pos |
| M149T261 | 7-Methyladenine | 149.0739 | 261 | 149.0701 | 25.49071 | BioDeepDB | pos |
| M150T271 | D-Cathinone | 150.0912 | 270.9 | 149.0841 | 0.825182 | BioDeepDB | pos |
| M153T79 | p-Hydroxyphenylacetic acid | 153.065 | 79 | 152.0473 | 5.312294 | BioDeepDB | pos |
| M154T311 | 3-Sulfinoalanine | 154.0043 | 311.4 | 153.0096 | 5.468756 | BioDeepDB | pos |
| M155T63 | Imidazol-5-yl-pyruvate | 155.047 | 63 | 154.0378 | 12.40914 | BioDeepDB | pos |
| M154T247 | 2-Pyrocatechuic acid | 154.0411 | 246.8 | 154.0266 | 4.008861 | BioDeepDB | pos |
| M154T178 | o-Hydroxylaminobenzoate | 154.0491 | 178.1 | 153.0426 | 3.878143 | BioDeepDB | pos |
| M157T157 | Suberic acid | 157.0836 | 157.1 | 174.0892 | 0.115633 | BioDeepDB | pos |
| M159T197 | 5-Acetamidovalerate | 159.0915 | 196.7 | 159.0895 | 12.57138 | BioDeepDB | pos |
| M162T99 | 3-Hydroxymethylglutaric acid | 162.058 | 98.8 | 162.0528 | 19.09922 | BioDeepDB | pos |
| M163T119 | L-erythro-4-Hydroxyglutamate | 163.0341 | 119.2 | 163.0481 | 28.76541 | BioDeepDB | pos |
| M166T58 | L-Methionine S-oxide | 166.0531 | 57.8 | 165.046 | 0.003529 | BioDeepDB | pos |
| M167T458 | Antiarol | 167.0701 | 458 | 184.0736 | 23.547 | BioDeepDB | pos |
| M168T117 | D-synephrine | 168.0911 | 116.6 | 167.0946 | 1.674923 | BioDeepDB | pos |
| M169T51 | (R)-4-Hydroxymandelate | 169.0582 | 51.4 | 168.0423 | 1.48287 | BioDeepDB | pos |
| M170T198 | Selenocysteine | 169.9773 | 197.6 | 168.9642 | 0.228097 | BioDeepDB | pos |
| M170T92 | Gallic acid | 170.0214 | 91.7 | 170.0215 | 0.588161 | BioDeepDB | pos |
| M171T44_1 | D-Glyceraldehyde 3-phosphate | 171.0057 | 43.9 | 169.998 | 0.441056 | BioDeepDB | pos |
| M172T332 | Tetrahydrodipicolinate | 172.0756 | 332.2 | 171.0532 | 1.99327 | BioDeepDB | pos |
| M173T70 | 2-Oxoarginine | 173.0785 | 69.9 | 173.08 | 0.18869 | BioDeepDB | pos |
| M175T54 | Amino acid(Arg-) | 175.1077 | 53.5 | 174.1117 | 1.407467 | BioDeepDB | pos |
| M178T48 | Coniferyl aldehyde | 178.0587 | 47.8 | 178.063 | 24.14934 | BioDeepDB | pos |
| M181T186 | L-Sorbose | 181.072 | 185.9 | 180.0634 | 7.312008 | BioDeepDB | pos |
| M182T76 | Hydroxyphenyllactic acid | 182.061 | 75.8 | 182.0579 | 17.02726 | BioDeepDB | pos |
| M199T191 | gamma-Glutamyl-beta-aminopropiononitrile | 199.0944 | 190.7 | 199.0957 | 6.529566 | BioDeepDB | pos |
| M185T110 | Vanylglycol | 185.0784 | 110.3 | 184.0736 | 13.37811 | BioDeepDB | pos |
| M185T297 | Sebacic acid | 185.1152 | 297.2 | 202.1205 | 0.020838 | BioDeepDB | pos |
| M188T217 | 3-Indoleacrylate | 188.0708 | 216.9 | 187.0633 | 0.000148 | BioDeepDB | pos |
| M189T411 | 7,8-Diaminononanoate | 189.1636 | 411.3 | 188.1525 | 17.47916 | BioDeepDB | pos |
| M207T82_2 | 4-(2-Aminophenyl)-2,4-dioxobutanoic acid | 207.0441 | 82.4 | 207.0532 | 0.419373 | BioDeepDB | pos |
| M190T332 | L-2-Amino-6-oxoheptanedioate | 190.0862 | 331.9 | 189.0637 | 0.516849 | BioDeepDB | pos |
| M195T458 | 3-Methyl-L-tyrosine | 195.1015 | 458.1 | 195.0895 | 0.040529 | BioDeepDB | pos |
| M195T615 | Neocnidilide | 195.1377 | 614.8 | 194.1307 | 0.104414 | BioDeepDB | pos |
| M199T76 | (3E)-3-[(1R,5R,6S)-5-Hydroxy-7-oxabicyclo[4.1.0]heptan-2-ylidene]-2-oxopropanoate | 199.0551 | 75.8 | 198.0528 | 12.72234 | BioDeepDB | pos |
| M202T199 | Thiabendazole | 202.0438 | 199 | 201.0361 | 2.098555 | BioDeepDB | pos |
| M202T257 | Spermine | 202.2165 | 257 | 202.2157 | 3.956156 | BioDeepDB | pos |
| M206T282 | Indolelactic acid | 206.0812 | 281.6 | 205.0739 | 0.002846 | BioDeepDB | pos |
| M209T415 | Anhalamine | 209.1171 | 414.5 | 209.1052 | 0.018642 | BioDeepDB | pos |
| M211T202 | 3-(3,4-Dihydroxy-5-methoxy)-2-propenoic acid | 211.0577 | 202.1 | 210.0528 | 10.52962 | BioDeepDB | pos |
| M221T437 | Caryophyllene alpha-oxide | 221.1899 | 437.4 | 220.1827 | 0.091796 | BioDeepDB | pos |
| M223T364 | Apiole | 223.0964 | 363.5 | 222.0892 | 0.34066 | BioDeepDB | pos |
| M229T307 | Traumatic Acid | 229.141 | 307.1 | 228.1362 | 8.818282 | BioDeepDB | pos |
| M234T448 | Confertifolin | 234.1619 | 447.7 | 234.162 | 0.427055 | BioDeepDB | pos |
| M237T358 | Procaine | 237.1651 | 357.8 | 236.1525 | 22.00595 | BioDeepDB | pos |
| M258T53 | Glycerophosphocholine | 258.1094 | 52.8 | 258.1106 | 1.36212 | BioDeepDB | pos |
| M247T380 | Mepivacaine | 247.1783 | 379.9 | 246.1732 | 7.849916 | BioDeepDB | pos |
| M249T186 | Pindolol | 249.1575 | 186.4 | 248.1525 | 0.010078 | BioDeepDB | pos |
| M276T339 | 4-Hydroxycinnamoylagmatine | 276.157 | 339.3 | 276.1586 | 5.793806 | BioDeepDB | pos |
| M261T53 | Glucose 6-phosphate | 261.0457 | 52.7 | 260.0297 | 27.84999 | BioDeepDB | pos |
| M265T309 | Tetracaine | 265.1882 | 308.7 | 264.1838 | 9.734365 | BioDeepDB | pos |
| M267T604 | 9-cis-Retinal | 267.2063 | 603.9 | 284.214 | 0.995486 | BioDeepDB | pos |
| M270T299 | 16-Oxopalmitate | 270.2169 | 298.6 | 270.2195 | 9.6219 | BioDeepDB | pos |
| M276T482 | Homatropine | 276.1587 | 481.7 | 275.1521 | 0.161784 | BioDeepDB | pos |
| M280T324 | Cyclopeptine | 280.1305 | 323.9 | 280.1212 | 2.203214 | BioDeepDB | pos |
| M297T477 | 3-Ketosphingosine | 297.2611 | 477.4 | 297.2668 | 19.17506 | BioDeepDB | pos |
| M283T395 | Adrenosterone | 283.169 | 394.7 | 300.1725 | 13.89276 | BioDeepDB | pos |
| M285T604 | Neoabietic acid | 285.2172 | 604 | 302.2246 | 0.119207 | BioDeepDB | pos |
| M286T424 | Piperine | 286.1434 | 424.1 | 285.1365 | 0.301395 | BioDeepDB | pos |
| M286T408 | 9-cis-Retinol | 286.2376 | 407.6 | 286.2297 | 27.59945 | BioDeepDB | pos |
| M290T421 | Dyclonine | 290.2085 | 420.9 | 289.2042 | 0.731031 | BioDeepDB | pos |
| M292T372 | Cyclopentolate | 292.1882 | 372.2 | 291.1834 | 7.94503 | BioDeepDB | pos |
| M295T216 | Aspartame | 295.1288 | 215.8 | 294.1216 | 0.00147 | BioDeepDB | pos |
| M296T50 | Dehypoxanthine futalosine | 296.0652 | 49.6 | 296.0896 | 1.74046 | BioDeepDB | pos |
| M301T152 | Salidroside | 301.1383 | 151.6 | 300.1209 | 7.917131 | BioDeepDB | pos |
| M308T358 | Dihydrocapsaicin | 308.219 | 358.2 | 307.2147 | 8.934102 | BioDeepDB | pos |
| M316T359 | Decanoyl-L-carnitine | 316.2482 | 358.6 | 315.241 | 0.00018 | BioDeepDB | pos |
| M318T462 | Chlorpromazine | 318.0978 | 462.3 | 318.0957 | 6.601743 | BioDeepDB | pos |
| M321T395 | 8,9-EET | 321.2507 | 395 | 320.2351 | 25.91123 | BioDeepDB | pos |
| M358T237 | Nalbuphine | 358.1988 | 236.8 | 357.194 | 0.998355 | BioDeepDB | pos |
| M347T363 | 21-Deoxycortisol | 347.2208 | 362.8 | 346.2144 | 0.015103 | BioDeepDB | pos |
| M353T384 | Prostaglandin I2 | 353.2291 | 383.9 | 352.225 | 3.106522 | BioDeepDB | pos |
| M353T412 | Lipoxin B4 | 353.2293 | 412.5 | 352.225 | 8.425122 | BioDeepDB | pos |
| M355T530 | Prostaglandin E1 | 355.2449 | 530 | 354.2406 | 8.377319 | BioDeepDB | pos |
| M364T556 | Sphinganine 1-phosphate | 364.2604 | 556 | 381.2644 | 9.427322 | BioDeepDB | pos |
| M376T689 | Docosatetraenoyl Ethanolamide | 376.3195 | 688.8 | 375.3137 | 0.046943 | BioDeepDB | pos |
| M377T254 | Estradiol benzoate | 377.2136 | 253.8 | 376.2038 | 6.691169 | BioDeepDB | pos |
| M379T619 | 3-Keto-4-methylzymosterol | 379.3382 | 618.8 | 396.3392 | 16.96112 | BioDeepDB | pos |
| M384T619 | Myriocin | 384.2861 | 619.2 | 401.2777 | 29.47244 | BioDeepDB | pos |
| M385T600 | Campestanol | 385.3778 | 600 | 402.3862 | 2.506631 | BioDeepDB | pos |
| M390T574 | 3-Geranylgeranylindole | 390.3115 | 574.5 | 389.3083 | 10.44294 | BioDeepDB | pos |
| M414T400 | 14alpha-Hydroxy-5beta-cholest-7-ene-3,6-dione | 414.2994 | 400.1 | 414.3134 | 0.275432 | BioDeepDB | pos |
| M423T657 | 24-Methylenecycloartanol | 423.3929 | 657 | 440.4018 | 3.462505 | BioDeepDB | pos |
| M424T619 | Alpha-Tocotrienol | 424.3323 | 618.9 | 424.3341 | 4.241958 | BioDeepDB | pos |
| M431T497 | Hecogenin | 431.3148 | 497.3 | 430.3083 | 0.430357 | BioDeepDB | pos |
| M432T236 | Gentamicin C1a | 432.279 | 236.1 | 449.2849 | 3.548634 | BioDeepDB | pos |
| M437T688 | FMLP | 437.1921 | 688.2 | 437.1984 | 5.156616 | BioDeepDB | pos |
| M482T543 | Antibiotic JI-20A | 482.322 | 543.4 | 481.2748 | 4.589032 | BioDeepDB | pos |
| M570T453 | 8,12-Diethyl-3-vinylbacteriochlorophyllide d | 570.2535 | 453.2 | 570.2481 | 9.469473 | BioDeepDB | pos |
| M570T490_2 | Avermectin B1b aglycone | 570.3538 | 489.7 | 570.3193 | 3.159025 | BioDeepDB | pos |
| M568T463_2 | Protoporphyrinogen IX | 568.3372 | 462.7 | 568.305 | 5.593325 | BioDeepDB | pos |
| M200T55 | 4-Fumarylacetoacetate | 200.0326 | 55.4 | 200.0321 | 2.499599 | MetaDNA | neg |
| M134T77 | 2-Phenylacetamide | 134.0599 | 76.6 | 135.0684 | 9.130164 | MetaDNA | neg |
| M528T260_1 | N-[(3a,5b,7a)-3-hydroxy-24-oxo-7-(sulfooxy)cholan-24-yl]-Glycine | 528.2616 | 259.7 | 529.2709 | 3.83142 | MetaDNA | neg |
| M128T50 | Pyroglutamic acid | 128.0336 | 49.6 | 129.0426 | 5.223172 | BioDeepDB | neg |
| M130T538 | Ketoleucine | 129.9747 | 538.2 | 130.063 | 0.007138 | BioDeepDB | neg |
| M113T84 | 2-Heptanone | 112.9834 | 84.1 | 114.1045 | 0.391331 | BioDeepDB | neg |
| M131T52 | Glutaric acid | 131.034 | 52 | 132.0423 | 0.328875 | BioDeepDB | neg |
| M113T200 | Dihydrouracil | 112.9835 | 199.7 | 114.0429 | 24.35604 | BioDeepDB | neg |
| M115T672 | trans-1,2-Cyclohexanediol | 114.9329 | 672.1 | 116.0837 | 0.991152 | BioDeepDB | neg |
| M115T58 | Fumaric acid | 115.0391 | 58.2 | 116.011 | 1.387096 | BioDeepDB | neg |
| M117T465 | Guanidoacetic acid | 116.9271 | 464.9 | 117.0538 | 1.339633 | BioDeepDB | neg |
| M117T672_2 | Betaine | 116.9271 | 672.1 | 117.079 | 1.226417 | BioDeepDB | neg |
| M117T74 | Succinic acid | 117.0545 | 74 | 118.0266 | 1.53664 | BioDeepDB | neg |
| M121T255 | Phenylethylamine | 121.0284 | 254.9 | 121.0891 | 0.260709 | BioDeepDB | neg |
| M122T58 | Nicotinic acid | 122.0237 | 57.9 | 123.032 | 0.877823 | BioDeepDB | neg |
| M124T51 | Taurine | 124.0064 | 51.3 | 125.0147 | 1.22192 | BioDeepDB | neg |
| M130T59 | L-Leucine | 130.0864 | 58.8 | 131.0946 | 1.092085 | BioDeepDB | neg |
| M132T48 | L-Aspartic acid | 132.0291 | 47.5 | 133.0375 | 0.126851 | BioDeepDB | neg |
| M135T299 | Phenylacetic acid | 134.8642 | 298.7 | 136.0524 | 3.531294 | BioDeepDB | neg |
| M135T685 | Phenyl acetate | 134.8935 | 684.8 | 136.0524 | 1.517582 | BioDeepDB | neg |
| M135T375 | Hypoxanthine | 134.9865 | 374.9 | 136.0385 | 14.21312 | BioDeepDB | neg |
| M137T58 | Urocanic acid | 137.0346 | 57.9 | 138.0429 | 0.051422 | BioDeepDB | neg |
| M142T215 | 1-Naphthylamine | 142.0653 | 214.9 | 143.0735 | 6.504051 | BioDeepDB | neg |
| M144T101 | Spermidine | 143.9145 | 100.8 | 145.1579 | 0.011268 | BioDeepDB | neg |
| M146T48 | L-Glutamic acid | 146.0449 | 47.6 | 147.0532 | 0.163658 | BioDeepDB | neg |
| M147T382 | Nornicotine | 147.0919 | 382.1 | 148.1 | 5.60194 | BioDeepDB | neg |
| M147T396 | trans-Cinnamate | 146.9649 | 396.4 | 148.0524 | 0.03256 | BioDeepDB | neg |
| M148T58 | L-Methionine | 148.0425 | 58 | 149.051 | 8.267896 | BioDeepDB | neg |
| M151T267 | D-Xylitol | 151.1119 | 266.6 | 152.0685 | 2.77468 | BioDeepDB | neg |
| M153T226 | Gentisic acid | 153.0193 | 225.6 | 154.0266 | 0.156843 | BioDeepDB | neg |
| M172T75_3 | N-Acetylleucine | 172.097 | 75.5 | 173.1052 | 2.022206 | BioDeepDB | neg |
| M159T72 | Oxoadipic acid | 158.9775 | 72.1 | 160.0372 | 2.159121 | BioDeepDB | neg |
| M180T58 | L-Tyrosine | 180.0653 | 57.6 | 181.0739 | 2.08161 | BioDeepDB | neg |
| M163T144 | 4-Hydroxycinnamic acid | 162.9817 | 144.3 | 164.0473 | 0.965138 | BioDeepDB | neg |
| M163T75 | Phenylpyruvic acid | 163.0391 | 74.9 | 164.0473 | 0.151946 | BioDeepDB | neg |
| M163T301 | L-Fucose | 163.112 | 301 | 164.0685 | 22.72915 | BioDeepDB | neg |
| M165T352 | 6-Methylmercaptopurine | 165.0195 | 352.2 | 166.0313 | 20.89462 | BioDeepDB | neg |
| M165T563 | Desaminotyrosine | 165.04 | 563.2 | 166.063 | 0.051775 | BioDeepDB | neg |
| M191T61 | Citric acid | 190.9541 | 60.6 | 192.027 | 14.77483 | BioDeepDB | neg |
| M175T43 | Guanidinosuccinic acid | 174.957 | 43 | 175.0593 | 11.85699 | BioDeepDB | neg |
| M179T52 | D-Mannose | 179.0549 | 52.4 | 180.0634 | 1.540205 | BioDeepDB | neg |
| M181T53 | Acetylcholine chloride | 180.9722 | 53.3 | 181.087 | 1.44173 | BioDeepDB | neg |
| M191T449 | 5-Hydroxyindoleacetic acid | 191.1071 | 448.7 | 191.0582 | 2.634592 | BioDeepDB | neg |
| M192T305 | Quinate | 192.0661 | 304.8 | 192.0634 | 14.05766 | BioDeepDB | neg |
| M199T526 | N-Acetylserotonin | 199.1695 | 525.6 | 218.1055 | 1.65554 | BioDeepDB | neg |
| M205T652 | Methyl jasmonate | 205.1589 | 652.1 | 224.1412 | 1.598193 | BioDeepDB | neg |
| M211T436 | Methyldopa | 211.1332 | 435.7 | 211.0845 | 0.49947 | BioDeepDB | neg |
| M213T554 | Tridecanoic acid | 213.1856 | 553.8 | 214.1933 | 0.104112 | BioDeepDB | neg |
| M218T75 | Pantothenic acid | 218.103 | 74.9 | 219.1107 | 0.034512 | BioDeepDB | neg |
| M221T495 | N-Acetyl-D-glucosamine | 221.1543 | 495.4 | 221.0899 | 0.462628 | BioDeepDB | neg |
| M242T553 | Thymidine | 242.1758 | 553.1 | 242.0903 | 0.337276 | BioDeepDB | neg |
| M243T39_1 | Cytidine | 242.9452 | 39.3 | 243.0855 | 7.262259 | BioDeepDB | neg |
| M243T57_2 | Uridine | 243.0621 | 56.8 | 244.0695 | 0.510158 | BioDeepDB | neg |
| M227T587 | Myristic acid | 227.2014 | 587.2 | 228.2089 | 0.608461 | BioDeepDB | neg |
| M239T52_2 | L-Cystine | 239.0152 | 51.9 | 240.0238 | 3.716144 | BioDeepDB | neg |
| M247T500 | Pyridoxal phosphate | 247.1702 | 499.9 | 247.025 | 0.447473 | BioDeepDB | neg |
| M249T53 | gamma-Glutamylcysteine | 248.9608 | 53.2 | 250.0623 | 2.444878 | BioDeepDB | neg |
| M250T566 | 2'-Deoxyadenosine | 250.1451 | 565.9 | 251.102 | 1.542101 | BioDeepDB | neg |
| M255T612 | Palmitic acid | 255.2319 | 612.1 | 256.2402 | 2.881685 | BioDeepDB | neg |
| M283T522 | Biochanin A | 283.17 | 522.2 | 284.0685 | 0.370733 | BioDeepDB | neg |
| M268T374 | Formononetin | 268.0698 | 374 | 268.0736 | 1.42158 | BioDeepDB | neg |
| M267T526 | Retinol | 267.1579 | 525.8 | 286.2297 | 0.818351 | BioDeepDB | neg |
| M289T55 | Epiandrosterone | 288.9538 | 55 | 290.2246 | 4.436719 | BioDeepDB | neg |
| M303T555 | Arachidonic acid | 303.2249 | 555.5 | 304.2402 | 26.46221 | BioDeepDB | neg |
| M289T525 | Dihydrotestosterone | 289.1659 | 525.5 | 290.2246 | 11.63131 | BioDeepDB | neg |
| M309T600_1 | Ribose 1,5-bisphosphate | 309.1739 | 600.2 | 309.9855 | 0.754594 | BioDeepDB | neg |
| M297T551 | Nonadecanoic acid | 297.244 | 551.1 | 298.2872 | 1.247793 | BioDeepDB | neg |
| M323T639 | CMP | 323.2208 | 638.6 | 323.0519 | 0.421777 | BioDeepDB | neg |
| M347T310 | dGMP | 347.1635 | 310.2 | 347.0631 | 20.60169 | BioDeepDB | neg |
| M340T563 | Fructose 1,6-bisphosphate | 340.2038 | 563.2 | 339.996 | 19.4697 | BioDeepDB | neg |
| M371T510 | Deoxycorticosterone acetate | 371.066 | 510.3 | 372.2301 | 0.138889 | BioDeepDB | neg |
| M377T406 | Ergocalciferol | 377.0578 | 405.9 | 396.3392 | 0.247746 | BioDeepDB | neg |
| M391T430 | Deoxycholic acid | 391.2859 | 430 | 392.2927 | 0.931723 | BioDeepDB | neg |
| M391T367 | Chenodeoxycholic acid | 391.2859 | 366.6 | 392.2927 | 1.216502 | BioDeepDB | neg |
| M397T446 | Daidzin | 397.2251 | 445.7 | 416.1107 | 3.45283 | BioDeepDB | neg |
| M425T486 | Lanosterin | 425.258 | 486 | 426.3862 | 0.153859 | BioDeepDB | neg |
| M448T327 | Glycochenodeoxycholic acid | 448.3075 | 326.8 | 449.314 | 0.010892 | BioDeepDB | neg |
| M431T414 | Genistin | 431.3185 | 414.4 | 432.1056 | 27.48035 | BioDeepDB | neg |
| M464T303 | Glycocholic acid | 464.3007 | 303 | 465.309 | 1.738226 | BioDeepDB | neg |
| M447T611 | Astragalin | 447.3487 | 610.5 | 448.1006 | 11.20163 | BioDeepDB | neg |
| M449T535 | Vitamin K1 | 449.0963 | 534.8 | 450.3498 | 3.188263 | BioDeepDB | neg |
| M472T550 | Maslinic acid | 472.3456 | 550.4 | 472.3553 | 20.53581 | BioDeepDB | neg |
| M107T243 | o-Cresol | 107.0491 | 243.2 | 108.0575 | 10.27566 | BioDeepDB | neg |
| M129T45 | Itaconic acid | 129.0183 | 45.4 | 130.0266 | 7.750838 | BioDeepDB | neg |
| M123T236 | 4-Methylcatechol | 123.0442 | 235.7 | 124.0524 | 7.509497 | BioDeepDB | neg |
| M130T231 | Beta-Guanidinopropionic acid | 130.0652 | 230.7 | 131.0695 | 22.88083 | BioDeepDB | neg |
| M134T58 | Adenine | 134.0462 | 57.8 | 135.0545 | 7.460114 | BioDeepDB | neg |
| M151T75 | Xanthine | 151.0252 | 74.7 | 152.0334 | 5.95927 | BioDeepDB | neg |
| M152T356 | Aminosalicylic Acid | 152.0352 | 355.6 | 153.0426 | 0.657742 | BioDeepDB | neg |
| M167T50_2 | Uric acid | 167.0209 | 50.4 | 168.0283 | 0.742422 | BioDeepDB | neg |
| M183T240 | 2,4-Dinitrophenol | 183.004 | 239.8 | 184.012 | 3.956198 | mzCloud | neg |
| M185T394 | (2R)-2-Hydroxy-3-(phosphonatooxy)propanoate | 184.9837 | 393.6 | 185.9929 | 10.40092 | BioDeepDB | neg |
| M189T57 | Carglumic acid | 189.0569 | 56.7 | 190.059 | 27.378 | BioDeepDB | neg |
| M194T81 | Salicyluric acid | 194.0454 | 80.9 | 195.0532 | 2.576717 | BioDeepDB | neg |
| M207T104 | L-Kynurenine | 207.0772 | 104.1 | 208.0848 | 1.448735 | BioDeepDB | neg |
| M253T592 | Palmitoleic acid | 253.2171 | 591.8 | 254.2246 | 0.806423 | MoNA | neg |
| M275T539 | Stearidonic acid | 275.2019 | 539 | 276.2089 | 0.726739 | BioDeepDB | neg |
| M295T600 | 9,10-Epoxyoctadecenoic acid | 295.2276 | 599.9 | 296.2351 | 0.758737 | BioDeepDB | neg |
| M279T600 | Bovinic acid | 279.2328 | 599.7 | 280.2402 | 0.444074 | BioDeepDB | neg |
| M283T355 | Glycitein | 283.0684 | 354.5 | 284.0685 | 25.35076 | BioDeepDB | neg |
| M293T448 | 13-L-Hydroperoxylinoleic acid | 293.2124 | 448.1 | 312.2301 | 2.353243 | BioDeepDB | neg |
| M311T600 | 9(S)-HPODE | 311.2232 | 599.5 | 312.2301 | 1.208136 | BioDeepDB | neg |
| M331T629 | Adrenic acid | 331.2642 | 629.1 | 332.2715 | 0.07245 | BioDeepDB | neg |
| M346T513 | Anandamide | 346.2746 | 513.2 | 347.2824 | 1.51325 | BioDeepDB | neg |
| M329T598 | Docosapentaenoic acid (22n-3) | 329.2478 | 598.3 | 330.2559 | 2.429781 | BioDeepDB | neg |
| M337T546 | 11,12-DiHETrE | 337.2364 | 545.9 | 338.2457 | 5.930558 | BioDeepDB | neg |
| M383T469 | Quetiapine | 383.1743 | 468.6 | 383.1667 | 19.83432 | BioDeepDB | neg |
| M409T447 | LysoPA(16_0_0_0) | 409.2356 | 446.7 | 410.2433 | 1.036078 | BioDeepDB | neg |
| M409T543 | Gamma-Tocotrienol | 409.3109 | 543.3 | 410.3185 | 0.732939 | BioDeepDB | neg |
| M499T412_2 | 1D-Myo-inositol 1,4,5,6-tetrakisphosphate | 498.9322 | 411.7 | 499.9287 | 21.59812 | BioDeepDB | neg |
| M558T556 | Cucurbitacin B | 558.3307 | 556.2 | 558.3193 | 20.41801 | BioDeepDB | neg |
| M158T49 | Allantoin | 158.045 | 49 | 158.044 | 6.327312 | BioDeepDB | neg |
| M116T158 | alpha-Ketoisovaleric acid | 116.0498 | 157.9 | 116.0473 | 21.54248 | MoNA | neg |
| M241T622 | Pentadecanoic acid | 241.2172 | 622.1 | 242.2246 | 0.431976 | MoNA | neg |
| M281T498 | Vaccenic acid | 281.2511 | 498.5 | 282.2559 | 8.803521 | MoNA | neg |
| M371T641 | (-)-Arctigenin | 371.1525 | 641.1 | 372.1573 | 6.671112 | MoNA | neg |
| M417T454 | Nimodipine | 417.1661 | 454.2 | 418.174 | 1.495807 | MoNA | neg |
| M463T450 | Estriol-16-Glucuronide | 463.2036 | 449.8 | 464.2046 | 13.47873 | MoNA | neg |
| M135T269 | o-Toluate | 135.0448 | 269.1 | 136.0524 | 2.399204 | mzCloud | neg |
| M179T380 | Isopropylparaben | 179.0707 | 380.2 | 180.0786 | 3.484657 | mzCloud | neg |
| M253T339 | Ketoprofen | 253.0834 | 339.1 | 254.0943 | 14.31939 | mzCloud | neg |
| M285T433 | Hexadecanedioate | 285.2074 | 433.3 | 286.2144 | 0.967717 | mzCloud | neg |
| M313T414 | 12,13-DHOME | 313.2391 | 413.6 | 314.2457 | 2.158096 | mzCloud | neg |
| M309T600_2 | 13(S)-HPOT | 309.2074 | 600.1 | 310.2144 | 0.892605 | mzCloud | neg |

*Abbreviations: mz (mass-to-charge ratio), rt (retention time), ppm (parts per million), pos/neg (positive/negative ionization mode).*
