# Supplementary material for: Integrated microbiome and metabolome profiling in prepubertal girls with vulvar lichen sclerosus
Source: Microbiol Spectr. 2026 Aug 27;14(10):e04058-25. doi: 10.1128/spectrum.04058-25 (PMC13689091; doi:10.1128/spectrum.04058-25)
Supplement: Supplementary file 7 [file spectrum.04058-25-s0007.docx]

**Miscellaneous File 6 Differential metabolites in VLS patients compared with the control group**

| **Metabolites** | **FC** | **log2FC** | ***P* value** | **-log10(*P* value)** | **FDR** | **VIP** |
| --- | --- | --- | --- | --- | --- | --- |
| Choline | 0.9 | -0.15 | 0.01367 | 1.86 | 0.059649 | 1.254626 |
| L-Serine | 2.05 | 1.04 | 1.8E-05 | 4.74 | 0.000411 | 1.972809 |
| Benzaldehyde | 1.1 | 0.14 | 1.61E-05 | 4.79 | 0.000381 | 2.093616 |
| m-Cresol | 1.65 | 0.73 | 0.000574 | 3.24 | 0.005554 | 1.66933 |
| Hydroquinone | 1.05 | 0.07 | 0.000346 | 3.46 | 0.003715 | 1.870718 |
| Creatinine | 0.84 | -0.25 | 0.00192 | 2.72 | 0.013571 | 1.426355 |
| N,N-Dimethyl-1,4-phenylenediamine | 1.28 | 0.36 | 0.023416 | 1.63 | 0.086433 | 1.105805 |
| (S)-1-Phenylethanol | 0.84 | -0.25 | 0.011981 | 1.92 | 0.054155 | 1.263142 |
| Benzoate | 1.43 | 0.51 | 9.28E-05 | 4.03 | 0.001422 | 1.93129 |
| 6-Hydroxyhexan-6-olide | 1.05 | 0.07 | 0.008597 | 2.07 | 0.042853 | 1.378639 |
| 6-Hydroxyhexanoic acid | 1.28 | 0.35 | 0.000884 | 3.05 | 0.00742 | 1.770027 |
| Creatine | 1.16 | 0.21 | 0.02001 | 1.7 | 0.076833 | 1.144714 |
| p-Aminobenzoic acid | 1.16 | 0.21 | 0.000872 | 3.06 | 0.00736 | 1.527116 |
| Acetylphosphate | 0.76 | -0.39 | 0.009637 | 2.02 | 0.046359 | 1.289206 |
| Pseudoephedrine | 1.39 | 0.48 | 1.22E-06 | 5.91 | 5.45E-05 | 2.254545 |
| Phenylglyoxylic acid | 0.89 | -0.17 | 0.009367 | 2.03 | 0.045542 | 1.278384 |
| D-Lyxose | 1.04 | 0.05 | 0.013382 | 1.87 | 0.058783 | 1.312549 |
| 3-Sulfinoalanine | 1.34 | 0.42 | 0.002042 | 2.69 | 0.014297 | 1.569165 |
| Suberic acid | 0.92 | -0.12 | 0.034774 | 1.46 | 0.113552 | 1.038485 |
| L-Carnitinamide | 1.3 | 0.38 | 0.000444 | 3.35 | 0.004532 | 1.633566 |
| Tolazoline | 1.36 | 0.44 | 0.003697 | 2.43 | 0.022715 | 1.531437 |
| Acetylcysteine | 1.17 | 0.23 | 0.004322 | 2.36 | 0.025473 | 1.386711 |
| L-Phenylalanine | 1.47 | 0.56 | 0.009403 | 2.03 | 0.045644 | 1.337145 |
| Quinolinic acid | 1.09 | 0.13 | 0.005527 | 2.26 | 0.030648 | 1.31147 |
| Selenocysteine | 1.15 | 0.21 | 0.001886 | 2.72 | 0.013382 | 1.558725 |
| Pyridoxine | 0.87 | -0.19 | 0.0279 | 1.55 | 0.096898 | 1.068402 |
| Azelaic acid | 1.33 | 0.41 | 1.21E-06 | 5.92 | 5.45E-05 | 2.222098 |
| 2-Oxoarginine | 1.09 | 0.13 | 0.004395 | 2.36 | 0.025801 | 1.404389 |
| Amino acid(Arg-) | 1.6 | 0.68 | 0.000104 | 3.98 | 0.001522 | 1.876238 |
| N-Methyltryptamine | 1.46 | 0.55 | 0.014956 | 1.83 | 0.063335 | 1.264956 |
| Shikimic acid | 1.14 | 0.18 | 0.027267 | 1.56 | 0.095412 | 1.085667 |
| Methyl beta-D-galactoside | 1.32 | 0.4 | 4.04E-05 | 4.39 | 0.000754 | 1.954137 |
| Theophylline | 1.42 | 0.5 | 0.000895 | 3.05 | 0.007466 | 1.582596 |
| L-Sorbose | 1.91 | 0.93 | 4.12E-05 | 4.39 | 0.000766 | 1.952562 |
| myo-Inositol | 1.55 | 0.63 | 4.23E-07 | 6.37 | 2.75E-05 | 2.401127 |
| Phosphorylcholine | 1.07 | 0.1 | 0.025352 | 1.6 | 0.090968 | 1.185765 |
| Phosphohydroxypyruvic acid | 1.15 | 0.2 | 0.004986 | 2.3 | 0.028423 | 1.317726 |
| Ecgonine | 1.2 | 0.26 | 0.001651 | 2.78 | 0.012102 | 1.547857 |
| 3-Indoleacrylate | 1.14 | 0.19 | 8.25E-05 | 4.08 | 0.001308 | 1.9285 |
| 7,8-Diaminononanoate | 0.69 | -0.53 | 3.74E-05 | 4.43 | 0.000722 | 1.944839 |
| D-Glucuronic Acid | 1.31 | 0.39 | 3.06E-05 | 4.51 | 0.000611 | 2.065763 |
| (3E)-3-[(1R,5R,6S)-5-Hydroxy-7-oxabicyclo[4.1.0]heptan-2-ylidene]-2-oxopropanoate | 1.6 | 0.68 | 7.21E-08 | 7.14 | 7.62E-06 | 2.525379 |
| Thiabendazole | 1.37 | 0.45 | 0.001186 | 2.93 | 0.009457 | 1.517592 |
| L-Tryptophan | 1.14 | 0.19 | 0.020087 | 1.7 | 0.077077 | 1.060881 |
| 3-(3,4-Dihydroxy-5-methoxy)-2-propenoic acid | 1.42 | 0.51 | 2.15E-05 | 4.67 | 0.000465 | 1.962535 |
| Caryophyllene alpha-oxide | 1.18 | 0.24 | 0.00978 | 2.01 | 0.046893 | 1.26547 |
| Porphobilinogen | 1.27 | 0.35 | 0.000535 | 3.27 | 0.005286 | 1.65762 |
| Procaine | 1.16 | 0.22 | 0.015274 | 1.82 | 0.064387 | 1.235447 |
| Lenticin | 0.79 | -0.34 | 0.007951 | 2.1 | 0.040312 | 1.324124 |
| Mepivacaine | 1.21 | 0.27 | 0.025505 | 1.59 | 0.091242 | 1.176403 |
| Pindolol | 1.37 | 0.46 | 1.53E-05 | 4.82 | 0.000373 | 2.030854 |
| OPEO | 0.83 | -0.26 | 0.005369 | 2.27 | 0.030156 | 1.334579 |
| Glycerophosphocholine | 1.68 | 0.75 | 0.02075 | 1.68 | 0.079008 | 1.113468 |
| 9-cis-Retinal | 1.5 | 0.59 | 2.03E-05 | 4.69 | 0.000451 | 1.971237 |
| 17a-Estradiol | 0.98 | -0.03 | 0.005413 | 2.27 | 0.030271 | 1.324938 |
| Linoleic acid | 0.75 | -0.42 | 0.000241 | 3.62 | 0.002802 | 1.824038 |
| (6Z)-Octadecenoic acid | 1.42 | 0.51 | 0.000237 | 3.63 | 0.002789 | 1.725468 |
| Adrenosterone | 0.83 | -0.27 | 0.006047 | 2.22 | 0.032764 | 1.383599 |
| Octadecanamide | 1.55 | 0.63 | 8.63E-06 | 5.06 | 0.00025 | 2.053601 |
| Neoabietic acid | 1.39 | 0.48 | 0.001362 | 2.87 | 0.010509 | 1.513515 |
| Cyclopentolate | 0.81 | -0.31 | 0.02629 | 1.58 | 0.093316 | 1.031258 |
| Sphingosine | 1.65 | 0.72 | 0.0001 | 4 | 0.001485 | 1.824985 |
| Arachidic acid | 1.56 | 0.64 | 1.21E-05 | 4.92 | 0.000318 | 2.067981 |
| Mycophenolate | 0.86 | -0.21 | 0.0042 | 2.38 | 0.025055 | 1.406904 |
| 8,9-EET | 0.88 | -0.19 | 0.027726 | 1.56 | 0.096502 | 1.212052 |
| Erucic acid | 1.35 | 0.43 | 0.000933 | 3.03 | 0.007718 | 1.556425 |
| Sucrose | 1.63 | 0.7 | 0.000282 | 3.55 | 0.003156 | 1.761294 |
| Cortexolone | 1.11 | 0.15 | 0.003295 | 2.48 | 0.020782 | 1.438123 |
| Lipoxin B4 | 1.17 | 0.23 | 0.010633 | 1.97 | 0.049707 | 1.33151 |
| Cholestane | 1.21 | 0.27 | 0.010283 | 1.99 | 0.048403 | 1.217862 |
| Nalbuphine | 1.2 | 0.27 | 0.000476 | 3.32 | 0.004775 | 1.678301 |
| Rosmarinic acid | 1.99 | 0.99 | 3.53E-06 | 5.45 | 0.000126 | 2.225314 |
| Thyrotropin releasing hormone | 1.84 | 0.88 | 1.29E-07 | 6.89 | 1.16E-05 | 2.384371 |
| Sphinganine 1-phosphate | 0.72 | -0.48 | 0.005798 | 2.24 | 0.031743 | 1.362808 |
| Lithocholic acid | 0.71 | -0.49 | 0.000183 | 3.74 | 0.002284 | 1.985897 |
| Docosatetraenoyl Ethanolamide | 1.54 | 0.62 | 0.001454 | 2.84 | 0.011004 | 1.499631 |
| Ergosta-5,7,22,24(28)-tetraen-3beta-ol | 1.23 | 0.3 | 0.004242 | 2.37 | 0.025175 | 1.318376 |
| Cornudentanone | 1.33 | 0.41 | 0.015864 | 1.8 | 0.065751 | 1.163625 |
| 3-Keto-4-methylzymosterol | 1.3 | 0.38 | 0.017164 | 1.77 | 0.069324 | 1.114743 |
| Campesterol | 1.18 | 0.24 | 0.011545 | 1.94 | 0.052776 | 1.199707 |
| Myriocin | 1.36 | 0.44 | 0.003382 | 2.47 | 0.021174 | 1.362969 |
| 7a-Hydroxy-5b-cholestan-3-one | 1.25 | 0.33 | 0.00084 | 3.08 | 0.007166 | 1.517275 |
| Campestanol | 0.7 | -0.51 | 0.023499 | 1.63 | 0.086544 | 1.058721 |
| Risperidone | 0.69 | -0.54 | 0.024582 | 1.61 | 0.089178 | 1.043635 |
| 3-Geranylgeranylindole | 1.85 | 0.88 | 0.000121 | 3.92 | 0.001708 | 1.786659 |
| Ursodeoxycholic acid | 0.86 | -0.22 | 2.43E-05 | 4.62 | 0.000514 | 2.049244 |
| beta-Sitosterol | 1.4 | 0.48 | 0.000162 | 3.79 | 0.002091 | 1.745815 |
| Stigmastanol | 1.32 | 0.4 | 0.00032 | 3.5 | 0.003464 | 1.662378 |
| 3beta,5beta-Ketotriol | 0.82 | -0.29 | 0.002475 | 2.61 | 0.016548 | 1.449819 |
| Antibiotic JI-20A | 0.8 | -0.33 | 0.007178 | 2.14 | 0.03719 | 1.352015 |
| 8,12-Diethyl-3-vinylbacteriochlorophyllide d | 0.75 | -0.42 | 0.000869 | 3.06 | 0.007354 | 1.524682 |
| Coproporphyrinogen III | 0.84 | -0.25 | 0.003696 | 2.43 | 0.022715 | 1.289301 |
| 2-Heptanone | 0.67 | -0.57 | 0.00139 | 2.86 | 0.021633 | 1.933549 |
| Guanidoacetic acid | 0.62 | -0.7 | 2.02E-05 | 4.69 | 0.001404 | 2.339307 |
| Betaine | 1.33 | 0.41 | 0.029686 | 1.53 | 0.145978 | 1.330106 |
| Beta-Guanidinopropionic acid | 1.17 | 0.23 | 0.039883 | 1.4 | 0.175804 | 1.225178 |
| o-Toluate | 0.85 | -0.23 | 0.005463 | 2.26 | 0.05177 | 1.519214 |
| Hypoxanthine | 0.87 | -0.21 | 0.028681 | 1.54 | 0.143518 | 1.392664 |
| 1-Naphthylamine | 1.35 | 0.43 | 0.038259 | 1.42 | 0.171058 | 1.250518 |
| Phenylpyruvic acid | 1.3 | 0.38 | 1.45E-06 | 5.84 | 0.000238 | 2.834858 |
| (2R)-2-Hydroxy-3-(phosphonatooxy)propanoate | 0.89 | -0.17 | 0.000386 | 3.41 | 0.009499 | 2.115981 |
| 5-Hydroxyindoleacetic acid | 0.85 | -0.23 | 0.000715 | 3.15 | 0.013673 | 1.893269 |
| 4-Fumarylacetoacetate | 0.91 | -0.14 | 0.004784 | 2.32 | 0.047457 | 1.700381 |
| Methyl jasmonate | 0.63 | -0.66 | 0.025262 | 1.6 | 0.132714 | 1.1581 |
| Methyldopa | 0.84 | -0.25 | 0.000704 | 3.15 | 0.013645 | 1.894203 |
| N-Acetyl-D-glucosamine | 0.89 | -0.16 | 0.000505 | 3.3 | 0.011393 | 1.968289 |
| Cytidine | 0.65 | -0.62 | 0.000147 | 3.83 | 0.005226 | 2.200634 |
| 2'-Deoxyadenosine | 0.89 | -0.16 | 0.010966 | 1.96 | 0.08069 | 1.176204 |
| Biochanin A | 0.79 | -0.35 | 0.013801 | 1.86 | 0.092436 | 1.388196 |
| 9,10-Epoxyoctadecenoic acid | 0.82 | -0.29 | 0.035223 | 1.45 | 0.16236 | 1.3884 |
| Nonadecanoic acid | 1.24 | 0.31 | 0.025754 | 1.59 | 0.133576 | 1.458349 |
| 12,13-DHOME | 0.75 | -0.42 | 0.007051 | 2.15 | 0.06083 | 1.476486 |
| CMP | 1.24 | 0.31 | 0.035753 | 1.45 | 0.163417 | 1.318575 |
| dGMP | 1.5 | 0.59 | 0.02604 | 1.58 | 0.134612 | 1.430594 |
| Deoxycorticosterone acetate | 0.35 | -1.52 | 0.001117 | 2.95 | 0.019096 | 1.799481 |
| Ergocalciferol | 0.88 | -0.19 | 0.004348 | 2.36 | 0.044673 | 1.589829 |
| Chenodeoxycholic acid | 0.88 | -0.18 | 5.04E-05 | 4.3 | 0.002581 | 2.275341 |
| Daidzin | 1.62 | 0.7 | 2.76E-05 | 4.56 | 0.001728 | 2.458633 |
| Gamma-Tocotrienol | 1.83 | 0.87 | 2.95E-05 | 4.53 | 0.001798 | 2.364004 |
| Lanosterin | 1.63 | 0.71 | 0.008278 | 2.08 | 0.067331 | 1.624643 |
| Astragalin | 0.87 | -0.2 | 0.023628 | 1.63 | 0.12708 | 1.136751 |
| Vitamin K1 | 1.43 | 0.52 | 0.000203 | 3.69 | 0.006362 | 2.208741 |
| N-[(3a,5b,7a)-3-hydroxy-24-oxo-7-(sulfooxy)cholan-24-yl]-Glycine | 1.19 | 0.26 | 0.047939 | 1.32 | 0.195348 | 1.323111 |

*Abbreviations: FC (Fold Change), log₂FC (log₂ Fold Change), P value (Probability value), FDR (False Discovery Rate), VIP (Variable Importance in Projection), VLS (Vulvar Lichen Sclerosus).*
