# Supplementary material for: Integrated microbiome and metabolome profiling in prepubertal girls with vulvar lichen sclerosus
Source: Microbiol Spectr. 2026 Aug 27;14(10):e04058-25. doi: 10.1128/spectrum.04058-25 (PMC13689091; doi:10.1128/spectrum.04058-25)
Supplement: Supplementary file 8 [file spectrum.04058-25-s0008.docx]

**Miscellaneous File 7 Pathways related to the differential metabolites screened by MetaboAnalyst**

| **Pathway id** | **Pathway name** | ***P* value** | **Compound name** |
| --- | --- | --- | --- |
| hsa00260 | Glycine, serine and threonine metabolism | 0.0002012 | L-Serine; L-Tryptophan; Choline; (2R)-2-Hydroxy-3-(phosphonatooxy)propanoate; Creatine; Guanidoacetic acid; Betaine; Phosphohydroxypyruvic acid |
| hsa05231 | Choline metabolism in cancer | 0.005536 | Choline; Phosphorylcholine; Glycerophosphocholine |
| hsa00100 | Steroid biosynthesis | 0.013382 | Lanosterin; beta-Sitosterol; Campesterol; Ergosta-5,7,22,24(28)-tetraen-3beta-ol; Ergocalciferol; 3-Keto-4-methylzymosterol |
| hsa02010 | ABC transporters | 0.021616 | L-Serine; L-Phenylalanine; Sucrose; Choline; myo-Inositol; N-Acetyl-D-glucosamine; Cytidine; 2'-Deoxyadenosine; Betaine; Methyl beta-D-galactoside |
| hsa00400 | Phenylalanine, tyrosine and tryptophan biosynthesis | 0.031829 | L-Tryptophan; L-Phenylalanine; Phenylpyruvic acid; Shikimic acid |
| hsa05230 | Central carbon metabolism in cancer | 0.038081 | L-Serine; L-Tryptophan; L-Phenylalanine; (2R)-2-Hydroxy-3-(phosphonatooxy)propanoate |
| hsa00591 | Linoleic acid metabolism | 0.041683 | Linoleic acid; 9,10-Epoxyoctadecenoic acid; 12,13-DHOME |
| hsa04978 | Mineral absorption | 0.048 | L-Serine; L-Tryptophan; L-Phenylalanine |
| hsa04071 | Sphingolipid signaling pathway | 0.0497384 | L-Serine; Sphingosine |
| hsa00600 | Sphingolipid metabolism | 0.049856 | L-Serine; Sphingosine; Sphinganine 1-phosphate |
