# Supplementary material for: Integrated microbiome and metabolome profiling in prepubertal girls with vulvar lichen sclerosus
Source: Microbiol Spectr. 2026 Aug 27;14(10):e04058-25. doi: 10.1128/spectrum.04058-25 (PMC13689091; doi:10.1128/spectrum.04058-25)
Supplement: Supplementary file 10 [file spectrum.04058-25-s0010.docx]

**Miscellaneous File 3 legends**

**Figure S1. Rarefaction curves of gut microbiota in control and VLS groups.
(A)** Rarefaction curves showing the relationship between sequencing depth and observed ASVs in VLS and control samples. **(B)** Boxplot comparing observed ASVs between control and VLS groups after rarefaction to 15,000 sequences per sample.
